# Disrupted valine metabolism and SCPC-mediated neurological toxicity in ECHS1 deficiency

**DOI:** 10.3389/fmolb.2026.1861051

**Published:** 2026-10-13

**Authors:** Meghan M. Eller, Madison A. Bunce, Mamta Verma, Raegan M. Adams, Xiaorong Fu, Alina P. Montalbano, Felix Nitschke, Shawn C. Burgess, Rachel M. Bailey

**Affiliations:** 1 Graduate School of Biomedical Sciences, University of Texas Southwestern Medical Center, Dallas, TX, United States; 2 Center for Alzheimer’s and Neurodegenerative Diseases, University of Texas Southwestern Medical Center, Dallas, TX, United States; 3 Center for Human Nutrition, University of Texas Southwestern Medical Center, Dallas, TX, United States; 4 Department of Pediatrics, University of Texas Southwestern Medical Center, Dallas, TX, United States; 5 Department of Biochemistry, University of Texas Southwestern Medical Center, Dallas, TX, United States; 6 Department of Pharmacology, University of Texas Southwestern Medical Center, Dallas, TX, United States

**Keywords:** ECHS1, epilepsy, mitochondrial disease, neurodevelopment, valine metabolism

## Abstract

**Background:**

ECHS1 Deficiency (ECHS1D) is a rare neurometabolic disorder caused by loss of function in the mitochondrial enzyme short-chain enoyl-CoA hydratase (ECHS1), leading to developmental delay, dystonia, seizures, and early mortality. Impaired ECHS1 activity disrupts valine catabolism, resulting in accumulation of reactive valine-derived intermediates associated with disease severity, though direct experimental evidence of neuronal toxicity and therapeutic modifiability remains limited. This study aimed to define the consequences of impaired ECHS1 activity, focusing on valine-derived intermediates and their impact on neuronal mitochondrial function.

**Methods:**

Wild-type and Echs1-deficient (Echs1D) mouse neuroblastoma (N2A) cells were utilized to investigate effects of patient-relevant valine intermediates S-(2-carboxypropyl)cysteine (SCPC), S-(2-carboxyethyl)cysteine (SCEC), and 2,3-dihydroxy-2-methylbutyric (DHMB) on mitochondrial function, oxidative stress, and cell viability. ECHS1D mice were used to quantify valine intermediates, evaluate pyruvate dehydrogenase complex (PDC) function, and assess seizure susceptibility under typical, excess, or restricted dietary valine intake.

**Results:**

In wild-type N2A cells, SCPC exposure induced dose-dependent cell toxicity and impaired mitochondrial respiration, while SCEC and DHMB exerted distinct mitochondrial effects. In contrast, Echs1D N2A cells displayed preserved mitochondrial fitness with premature neuronal differentiation, indicating cell-type-specific metabolic adaptations. ECHS1D mice accumulated SCPC, which was exacerbated by excess dietary valine and associated with reduced pyruvate dehydrogenase complex activity, and worsened seizure susceptibility. Valine restriction initiated during adolescence failed to reduce SCPC or improve seizure outcomes and instead increased SCEC levels.

**Conclusion:**

These studies demonstrate that accumulation of ECHS1D-specific valine intermediates, particularly SCPC, contributes to mitochondrial dysfunction and disease severity in ECHS1D. Dietary valine modulation during adolescence showed limited therapeutic benefit and induced broader metabolic perturbations, underscoring the need for improved therapeutic strategies.

## Introduction

ECHS1 Deficiency (ECHS1D) is a rare neurometabolic pediatric disorder characterized by developmental delays and regressions, hypotonia, dystonia, and often early mortality (Ozlu et al., 2022). Short-chain enoyl-CoA hydratase (ECHS1) is a nuclear-encoded mitochondrial enzyme responsible for the breakdown of valine, with secondary roles in the catabolism of leucine, isoleucine, and short-chain fatty acids (Burgin and McKenzie, 2020). Loss of function variants that impact mitochondrial localization, substrate binding, or protein stability result in ECHS1D. There is a wide phenotypic spectrum in patients, and although no direct genotype-phenotype correlation has been established, variants that result in near-complete or complete loss of enzymatic activity are associated with more severe disease outcomes (Masnada et al., 2020).

Patients with ECHS1D frequently exhibit accumulation of valine-derived metabolic intermediates arising from the ECHS1-specific substrates methacrylyl-CoA and acryloyl-CoA (Kuwajima et al., 2021), whereas metabolites from other branched-chain amino acids (BCAAs; leucine and isoleucine) or from fatty acid β-oxidation are often not elevated. This supports impaired valine metabolism as a primary driver of metabolic dysfunction in ECHS1D. Notably, increased levels of cysteine and cysteamine conjugates of the valine intermediate methacrylyl-CoA and acryloyl-CoA in patient samples are correlated with earlier disease onset and more severe clinical phenotypes (Liu et al., 2025). These metabolites are expected to induce cellular toxicity (Brown et al., 1982; Kuwajima et al., 2021; Peters et al., 2014), suggesting a mechanistic link between metabolite accumulation and disease progression. However, despite these clinical correlations, direct experimental evidence assessing the toxicity of these ECHS1D-specific valine metabolites remains limited.

Approximately half of ECHS1D patients present with secondary deficiency in the pyruvate dehydrogenase complex (PDC), which is hypothesized to be caused by toxic metabolite accumulation (Bedoyan et al., 2017). Inhibition of this complex is associated with mitochondrial dysfunction, due to decreased pyruvate oxidation which limits TCA flux (Patel et al., 2012). As such, ECHS1D cell models and patient samples display impaired mitochondrial respiration, defects in OXPHOS complex assembly, and alterations in production of reactive oxygen species (ROS) (Burgin et al., 2023; Burgin et al., 2022; Li et al., 2025). These assessments, however, have been limited to fibroblasts and epithelial-like cells; thus, our knowledge of neuronal mitochondrial health in ECHS1D remains largely unknown.

Recent therapeutic interventions have tested the utility of valine restricted diets to reduce toxic metabolite accumulation and improve disease outcomes. In an ECHS1D *Drosophila* model, valine restriction was sufficient to extend larval survival, although the degree of restriction tested also impaired development in wild-type (WT) flies (Mele et al., 2025). In patients, valine-restricted regimens indicate benefit in some patients, with modest improvements reported in clinical and metabolic parameters, though responses have been highly variable (Abdenur et al., 2020; Kuwajima et al., 2021; Sato-Shirai et al., 2021; Shayota et al., 2019). The absence of standardized dietary protocols and limited evaluation in larger well-characterized cohorts limits the interpretation of these clinical findings. Together, these observations underscore the need for systematic studies to define levels of valine intake that mitigate disease pathology while preserving normal growth and development.

In this study, we define how impaired ECHS1 activity disrupts mitochondrial function in neuronal cells using both pharmacologic exposure to ECHS1D-specific metabolites and genetic loss of Echs1. We provide direct evidence of cellular toxicity from patient-relevant valine-derived intermediates and report cell-type specific effects of Echs1 loss, as neuroblastoma knockdown cells display increased mitochondrial fitness accompanied by premature neuronal differentiation. Extending these findings, we detect toxic valine-derived intermediates in a previously characterized ECHS1D mouse model (Eller et al., 2025) and demonstrate that increased metabolite accumulation is associated with worsened seizure susceptibility. Finally, we evaluate a valine restriction paradigm in this mouse model and find that decreased valine intake alters metabolic pathways without improving seizure susceptibility. Together, these results support the role of impaired valine metabolism as a key contributor to disease progression and underscore the need for therapeutic strategies beyond dietary modulation.

## Methods

### Cell culture and treatments

Mouse neuroblastoma cells (N2As) were cultured in Dulbecco’s Modified Eagle Medium (DMEM) media supplemented with 10% fetal bovine serum (FBS), 1% Gluta-Max, 50 units/mL penicillin and 50 μg/mL streptomycin at 37 °C/5% CO_2_.

For treatment with valine intermediates, S-(2-carboxypropyl)cysteine (SCPC) (TargetMol, TN7107), S-(2-carboxyethyl)cysteine (SCEC) (A2B Chem, AF72480), and 2,3-dihydroxy-2-methylbutyric (DHMB) (A2B Chem, AW04224) were dissolved in Opti-MEM and treated at concentrations of 5–25 mM with Lipofectamine 2000 (Thermo Scientific #11668019). Lipofectamine was used to ensure cellular entry of these negatively charged metabolites. Wells treated with Lipofectamine alone were used as controls. 48 h after the indicated treatments, cells were harvested for subsequent analyses including viability and mitochondrial assessments.

For siRNA treatment, cells were transfected via Lipofectamine 2000 with 40 nM of scrambled siRNA control (Sigma # SIC001) or siRNA targeting mouse Echs1 (Sigma # PDSIRNA2D) dissolved in Opti-MEM. After 48 h of treatment, cells were harvested for subsequent analyses.

### Generation of Echs1D cell line

Guide RNA sequences against the mouse *Echs1* sequence were generated using the Brie library (Doench et al., 2016) and cloned into the px330-mcherry Cas9 plasmid that was a gift from Jinsong Li (Addgene plasmid #98750; http://n2t.net/addgene:98750; RRID: Addgene_98750) (Wu et al., 2013). WT N2As were transfected with plasmid containing one of the four guide options, and mcherry-positive single cells were obtained using fluorescence-activated cell sorting (FACS). Single cells were expanded and screened for CRISPR-mediated Echs1 knock-out via Western blot, with Sanger sequencing that confirmed heterozygous gene editing. A single clone of Echs1 deficient (Echs1D) N2A was used for experiments, and WT N2As of similar passage number were used as control. Echs1D and WT N2A were used up to passage 4.

### Cell viability

Cells were removed via trypsinization and stained with trypan blue. Viability counts were performed using an automated cell counter (Biorad, #1450102).

### Flow cytometry

For all flow cytometry experiments, WT and Echs1D N2A cells were plated in a 96-well plate at a density of 20,000 cells per well and provided respective treatments as described. Cells were transferred to round bottom plates and treated with 1uM MitoTracker Green FM (Thermo Fisher, M46750), 1uM MitoTracker Red FM (Thermo Scientific, #M22425), 1uM MitoSOX Red (Thermo Scientific, #M36008) or costained with 1uM of MitoTracker Red FM and 1uM MitoTracker Green FM. Immediately following staining, flow cytometry was performed on an Attune Flow Cytometer, and analysis was performed on FlowJo software (version 10.10.0). Cells were gated to exclude debris and only include singlets. Compensation was performed so that all unstained wells had less than 1% of cells had positive fluorescent signal.

### Seahorse extracellular flux assay

For analysis of mitochondrial respiration, the Seahorse XF96 Cell Mito Stress test was performed. WT and Echs1D N2A cells were plated in Seahorse XF96 Cell Culture Microplate at a density of 6,000 cells per well. At 24 h after plating, cells were treated and the stress test was performed 48 h later as described (Gu et al., 2021).

### Western blotting

WT and ECHS1D N2As were collected via trypsinization, washed in PBS and stored at −80 °C. For protein extraction, cell pellets were thawed on ice and lysed in NP-40 buffer (50 mM Tris-HCl, pH 7.4; 50 mM NaCl; 0.5% NP-40) supplemented with Protease Inhibitor Cocktail (ThermoFisher, #A32955). Lysates were incubated on ice for 10 min, sonicated four times, and centrifuged at 12,000 rpm for 15 min at 4 °C. Supernatants containing soluble proteins were collected and either stored at −80 °C or processed immediately for protein quantification. Protein concentration was determined using the Pierce BCA Protein Assay Kit (Thermo Fisher Scientific, #23225). Equal amounts of protein were resolved by on 4%–20% Mini-PROTEAN TGX Precast Protein Gels (Bio-Rad, #4561093) using 10× Tris/Glycine/SDS running buffer (Bio-Rad, #1610772). Proteins were then transferred to PVDF membranes using the Trans-Blot Turb RTA Mini PVDF Transfer Kit (Bio-Rad, #1704272). Membranes were blocked in 5% nonfat dry milk prepared from Blotting-Grade Blocker (Bio-Rad, #1706404).

Blots were incubated with rabbit anti-ECHS1 (1:1,000; Abnova, #H00001892-D01P) or rabbit anti-NeuN (1:1,000; Sigma, #ABN78) followed by rabbit peroxidase secondary antibody (1:10,000; Jackson Immuno #111-035-144). Protein loading was normalized to GAPDH (primary 1:10,000, Meridian #H68504M; secondary 1:10,000 Jackson Immuno #115-035-146). Quantification was performed using ImageJ (Version 2.1.0).

### OXPHOS native gel

WT and Echs1D N2A cells were trypsinized and 1.6E6 cells were collected and lysed in 50 μg/mL digitonin solution in dPBS with protease inhibitor (ThermoFisher, #A32955). Mitochondria were isolated through centrifugation as previously described (Wieckowski et al., 2009) and protein content was measured with the Pierce 660 nm Protein Assay (ThermoFisher, #22660). 25 μg of protein was solubilized in sample buffer (Invitrogen BN2003) containing 5% digitonin (Sigma D141) and centrifuged at 15,000 × g for 10 min. G-250 coomassie additive (Invitrogen BN2004) was added to the supernatant. Protein was loaded on a 3%–12% gel (Invitrogen BN10001B0X) and run using Invitrogen buffers (BN2002, BN2001) as described (Nishihara et al., 2021). The gel was stained with Coomassie to image the NativeMark ladder (Thermofisher, #LC0275), then destained and transferred onto a PVDF membrane. The membrane was fixed (Schägger, 2006) and blocked in 5% BSA in TBST, incubated in OxPhos cocktail (1:5,000; Abcam, ab110413) and then mouse peroxidase secondary antibody (Jackson Immuno #115-035-146) in 5% BSA in TBST. Quantification was performed using ImageJ (Version 2.1.0).

### Immunofluorescence

WT and Echs1D cells were plated on sterile coverslips and fixed with 4% PFA after 72 h in culture. Cells were permeabilized using 0.1% Triton X-100 and immunocytochemistry was performed using standard techniques. Primary antibody used was anti-β-Tubulin (1:1,000, Cat#) and secondary antibody was goat anti-mouse 594 (1:1,000, Invitrogen #A11005). Counter staining with DAPI (1:1,000) was performed prior to cover-slipping with Aqua Poly-mount.

### Imaging and analysis

Images of WT and Echs1D cells were taken on a Zeiss LSM780 inverted +2-Photon laser scanning microscope (15 images per cell line). All images were processed in ImageJ and analyzed in Ilastik for branching and nuclei size using pixel classification. For pixel classification, all images were used for training and all available features were selected. To segment for branching, tubulin was annotated. To segment for nuclei, DAPI was annotated. The probabilities generated in pixel classification were then used in object classification along with their respective images. A threshold and size filter were applied to exclude objects too large or small to be nuclei or branches. Once objects were classified, branch length was defined by exporting the average branch length in pixels of objects classified as branches for each image. The average nuclei size was defined by exporting the average object area in pixels of objects classified as nuclei for each image.

### Experimental animals

All procedures were performed in accordance with protocols approved by the Institutional Animal Care and Use Committee of the University of Texas Southwestern Medical Center (UT Southwestern), an AAALAC-accredited facility. Breeders and experimental mice were maintained under controlled environmental conditions, 12-h light-dark cycles, and provided food and water *ad-libitum*. ECHS1D mice were generated at the Jackson Laboratory on a C57BL/6J background using CRISPR/Cas9 genome editing as previously described (Eller et al., 2025). WT littermates were used as controls and order of testing or sample collection was randomized among genotypes for all experiments. Equal numbers of male and female mice were included in all experimental groups, and data from both sexes were pooled after confirming that there were no significant sex-dependent differences.

### Customized diets

For valine supplementation, a custom diet containing 3.8% valine was formulated by Inotiv using their standard 2016 diet as a base. Valine chow was provided to breeders and their weaned litters. Standard 2016 diet (contains 0.8% valine) was used as control. For valine restriction, a custom diet containing 0.2% valine was formulated by Inotiv using an amino acid defined diet as a base. All chows were stored at 4 °C and replaced weekly. Complete diet compositions are provided in Supplementary Table S1.

### PTZ seizure induction

The pentylenetetrazol (PTZ) kindling model was performed as described (Shimada and Yamagata, 2018). Briefly, mice received an intraperitoneal (i.p.) injection of 30 mg/kg PTZ dissolved in sterile saline every other day for a total of 12 injections. Mice were observed for 30 min following injection and seizure severity was scored by a blind observer using a standard Racine scale.

### Focused microwave brain fixation and harvest

At 3 months of age, mice were euthanized using a 5 kW Muromachi Microwave Fixation System (MMW-05, Muromachi Kikai Co., Ltd., Tokyo, Japan) to preserve the metabolic profile by rapidly inactivate enzymes involved in the metabolism of metabolites and neurotransmitters (Juras et al., 2023; Nitschke et al., 2025). Briefly, the unanesthetized mouse was restrained in an animal holder where the head was positioned in a water-jacketed cone (WJM-28, Muromachi Kikia Co. Ltd.) and the body immobilized using a plunger. The animal holder was carefully positioned in the applicator head of the MMW-05 unit and a high-energy microwave (5 kW) was used to irradiate the head of the mouse for 1.40 s. The microwave-fixed brain was immediately excised and frozen in liquid nitrogen.

### Measurement of nucleotides and short chain Acyl-CoAs

Ion-pairing reverse-phase liquid chromatography ionization-tandem mass spectrometric was used to quantify adenine nucleotides and short-chain acyl-CoAs as previously reported (Fu et al., 2019). Frozen brain samples were immediately spiked with stable-isotope-labeled ATP, acetyl-CoA, and malonyl-CoA internal standards. Approximately 50 mg of frozen tissue was homogenized in 500 μL of 0.4 M HClO_4_ (PCA) containing 0.5 mM EGTA, and the tissue remained on ice for 10 min before being centrifuged at 14 000 g at 4 °C for 10 min. The supernatant (400 μL) was neutralized with 136 μL of 0.5 M K_2_CO_3_, and the precipitate (KClO_4_) was removed by centrifugation at 14,000 × g at 4 °C for 24 min. Neutralized supernatants were subjected to LC-MS/MS analysis on the same day. Analysis was done on an API 5000 triple quadrupole LC-MS/MS mass spectrometer (Applied Biosystems/Sciex Instruments) in positive electrospray ionization mode. The mass spectrometer was equipped with a Shimadzu LC-20AD liquid chromatograph and a SIL-20ACHT auto sampler. A reverse-phase C18 column (Atlantis T3 Column, 150 × 2.1 mm, 3 µm) was used with LC mobile phase consisting of water/methanol (95:5, v/v) with 4 mM dibutylamine acetate (eluent A), and water/acetonitrile (25:75, v/v; eluent B). Multiple-reaction monitoring (MRM) of MS/MS was used to detect each analyte and its stable isotope.

### Measurement of amino acids

Frozen brain samples were spiked with stable isotope labeled amino acid internal standards (Isotec) and quantified using LC-MS/MS as we previously reported (Deja et al., 2024). Approximately 20 mg of tissue was homogenized in 4% PCA, and the supernatant was neutralized with 0.5 M K_2_CO_3_, followed by precipitation by centrifugation at 14 000 g at 4 °C for 10 min. The supernatants were then removed and dried under N_2_. The dried extracts were derivatized as described previously (Fu et al., 2019). Amino acids were separated on a reverse phase C18 column (Xbridge, Waters, Milford, MA; 150 × 2.1 mm, 3.0 μm) with gradient elution and detected using the MRM mode by monitoring specific transitions under positive electrospray on Triple Quad 5500+ LC/MS/MS mass spectrometer (Applied Biosystems/Sciex Instruments). Quantification was done by comparing individual ion peak areas to that of an internal standard.

### Measurement of valine intermediates

Cellular and blood extracts were analyzed by LC-MS by the Children’s Research Institute Metabolomics Core at UT Southwestern. SCPC (TargetMol, TN7107), SCEC (A2B Chem, AF72480), and DHMB (A2B Chem, AW04224) were used as reference standards for peak identification based on retention time and mass spectral characteristics. Total ion chromatogram (TIC)-normalized metabolite signals were calculated and represented as relative to untreated controls.

### Statistics

One-way ANOVA with Dunnett’s multiple comparisons was used to compare the means of three or more independent groups. Two-way ANOVA was used for multiple group comparisons, and Fisher’s protected LSD was used to identify genotype or diet effects only when the initial ANOVA for that variable was significant. Mixed effects models with Tukey’s multiple comparisons were used for repeat measure multiple group comparisons. All ANOVA statistics are presented in Supplementary Material. Unpaired, two-tailed Student’s t-test was used for comparisons between two groups. Geisser-Greenhouse’s epsilon correction was used if the estimated epsilon was <0.75. Survival differences were analyzed using the log rank test. Statistical significance was set at p ≤ 0.05. Outliers were determined using Grubbs’ test. Statistical analysis and graphing were done using GraphPad Prism software (v9.5.0; GraphPad Software). Values are expressed as mean±SEM. Statistical analyses results are included in Supplementary Tables S1–S11.

## Results

### Cellular toxicity of valine intermediates

Accumulation of reactive, cysteine- and cysteamine-conjugated valine-derived intermediates has been implicated in ECHS1D pathology (Figure 1A). To determine whether elevated levels of ECHS1D-associated valine intermediates impact cell viability, WT mouse neuroblastoma cells (N2A) were treated with a pool of S-(2-carboxypropyl) cysteine (SCPC), S-(2-carboxyethyl) cysteine (SCEC), and 2,3-dihydroxy-2-methylbutyric acid (DHMB), which are reported to accumulate in patients. This cell line was selected to provide a neuronal context, given the prominent neurological manifestations of ECHS1D (Marti-Sanchez et al., 2021; Ozlu et al., 2022), and the intermediates tested were selected based on commercial availability. After 48 h of treatment, cell viability was significantly reduced relative to control, supporting the toxic effects of these metabolites (Figure 1B). To determine the effects of individual intermediates, we treated cells with increasing concentrations of SCPC, SCEC, or DHMB alone. At high concentrations (25 mM), SCPC markedly reduced cell viability by ∼75% (Figure 1C). In contrast, SCEC had no effect at the concentrations tested; however, limited solubility restricted the maximum dose to 15 mM (Figure 1D). DHMB exposure produced a more modest effect, with high concentrations resulting in a ∼20% reduction in viability (Figure 1E). Notably, the reduction in cell survival observed with low concentrations of each metabolite in the pooled mixture suggests a synergistic effect, as substantially higher concentrations of individual metabolites were necessary to reduce survival.

**FIGURE 1:**
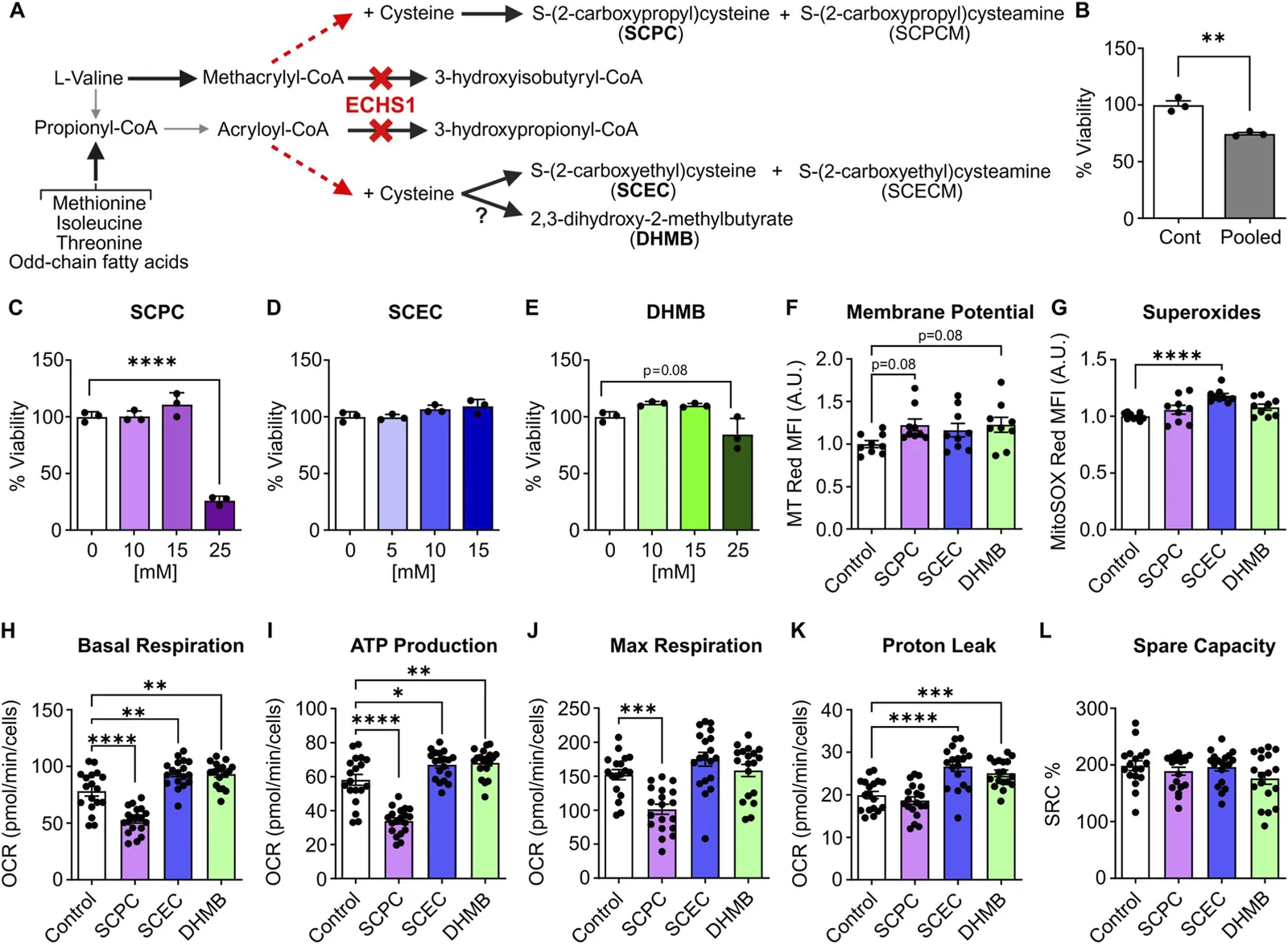
Valine intermediate SCPC promotes toxicity and impairs mitochondrial function in N2As. **(A)** Schematic of cysteine conjugated valine intermediates that accumulate following ECHS1 loss. Modified from Peters et al., (2014). **(B)** WT N2A cells were treated with Lipofectamine only (Cont) or a pool of SCPC, SCEC, and DHMB mixed with Lipofectamine (pooled). Individual metabolite concentrations were 5 mM, for a total pooled concentration of 15 mM. **(C–E)** WT N2A cells were treated with increasing concentrations of SCPC, SCEC, or DHMB, and percent viability was quantified. Each dot represents an individual well. **(F, G)** Cells were treated with 10 mM of SCPC, SCEC, or DHMB, and stained with MitoTracker Red **(F)** or MitoSOX **(G)** to quantify mitochondrial membrane potential and superoxide formation, respectively. Values are mean fluorescence intensity (MFI) relative to control. Data graphed are from three independent experiments; each dot represents an individual well. **(H–L)** Following treatment with 10 mM of SCPC, SCEC, or DHMB, oxygen consumption rate was measured using an XF96 Seahorse Analyzer. Basal respiration **(H)**, ATP production **(I)**, maximum respiration **(J)**, proton leak **(K)**, and spare capacity **(L)** were calculated. Data graphed are from three independent experiments; each dot represents an individual well. **(B)** Student’s t-test, **p < 0.01; **(C–L)** One-way ANOVA with Dunnett’s multiple comparison, *p < 0.05, **p < 0.01, ***p < 0.001, ****p < 0.0001. Full ANOVA results are shown in Supplementary Table S4.

For treatment with metabolic intermediates, lipofectamine was used to facilitate cellular uptake, as these compounds are expected to be negatively charged at physiological pH. To determine whether lipofectamine was necessary for intermediate delivery though, cells were treated with SCPC alone or in combination with lipofectamine (Supplementary Figure S1). Lipofectamine did not significantly exacerbate SCPC-induced toxicity compared with SCPC treatment alone (Supplementary Figure S1A). Interestingly, relative intracellular SCPC levels assessed 48 h after treatment were significantly lower in cells treated with SCPC and lipofectamine than in cells treated with SCPC alone (Supplementary Figure S1B). Although this finding could indicate that lipofectamine impaired SCPC uptake, the similar effects on cell viability with and without lipofectamine raise the alternative possibility that lipofectamine enhanced initial SCPC uptake, followed by greater clearance by the 48-h time point. While additional studies will be required to define the effect of lipofectamine on intracellular exposure, they do support that valine-derived intermediates are sufficient for toxicity in neuronal-like cells.

Impaired mitochondrial respiration has been reported in fibroblasts from more severely affected patients, who also exhibit greater accumulation of valine metabolites (Burgin and McKenzie, 2020; Kuwajima et al., 2021; Yamada et al., 2015). To test whether excess valine intermediates directly alter mitochondrial function, cells were treated with a sub-toxic concentration (10 mM) of each metabolite and assessed using MitoTracker and MitoSOX staining to quantify mitochondrial membrane potential and superoxide production, respectively (Figures 1F,G). SCPC and DHMB treatment resulted in mild increases in membrane potential, although this was not associated with increased superoxide formation. In contrast, SCEC significantly increased superoxide production, which is often associated with altered mitochondrial respiration (Figures 1F,G) (Brand, 2016).

To directly quantify respiratory function, a Seahorse extracellular flux assay was then performed following intermediate metabolite treatment (Figures 1H–L). Consistent with cell viability outcomes, only SCPC significantly impaired mitochondrial function, as indicated by reduced basal respiration, ATP-linked respiration, and maximum respiration (Figures 1H–J). In contrast, SCEC and DHMB treatment enhanced ATP production despite increased proton leak relative to untreated controls (Figures 1I–K). Notably, spare respiratory capacity was not significantly affected by any treatment condition (Figure 1L).

Collectively, these results reveal differential effects of ECHS1D-associated metabolites on cell viability and mitochondrial function and provide evidence that high concentrations of SCPC can cause neuronal toxicity, alone or in combination with SCEC and DHMB.

### Mitochondrial function in Echs1D cells

Having established that excess valine-derived intermediates are sufficient to impair mitochondrial function in WT N2A cells, we next sought to determine the effects of Echs1 deficiency on mitochondrial function in a neuronal cell model. To this end, we used CRISPR-Cas9 to generate a monoclonal Echs1-deficient (Echs1D) N2A cell line with no detectable Echs1 protein expression via Western blot analysis (Figure 2A).

**FIGURE 2:**
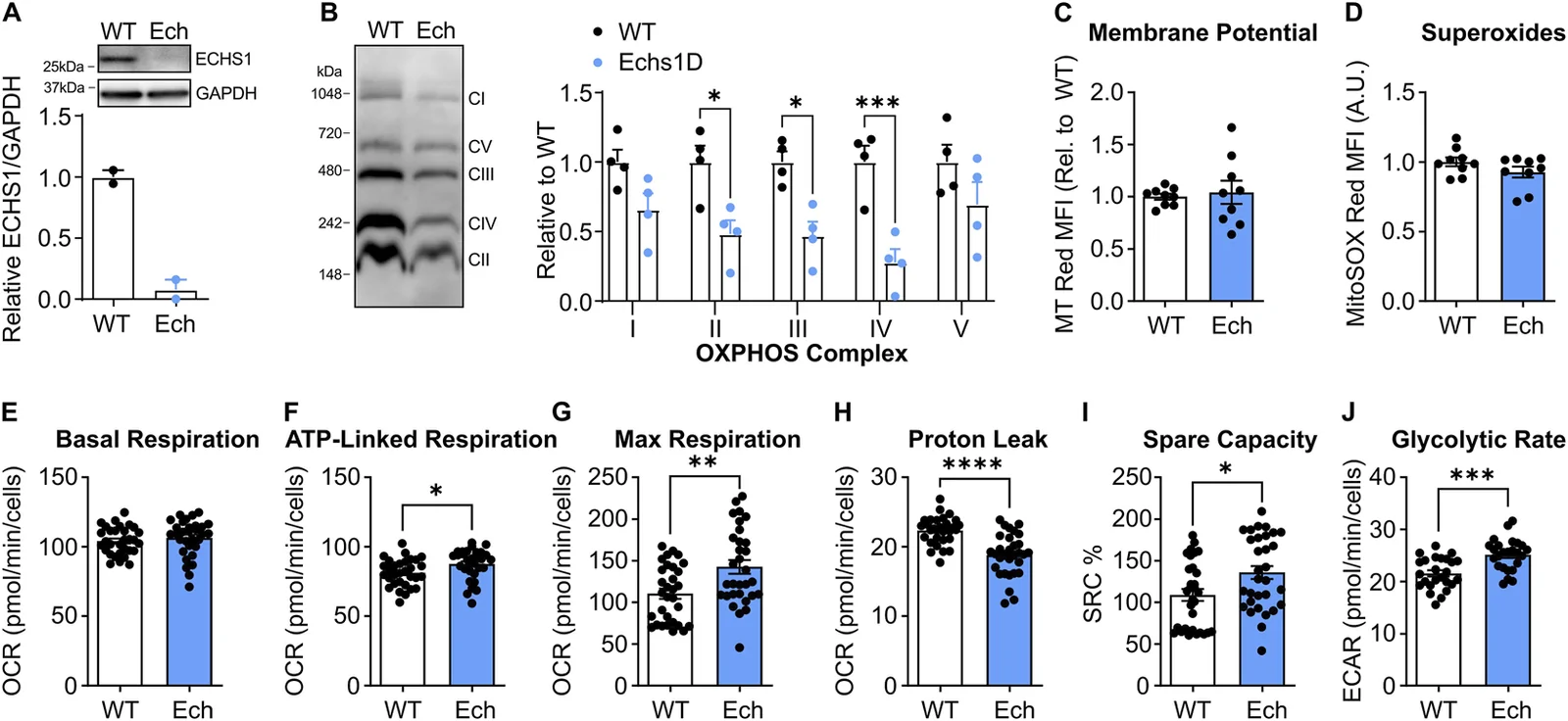
Echs1D N2As have reduced OXPHOS complex assembly but preserved mitochondrial function. **(A)** Echs1-deficient (Echs1D; Ech) cells were generated and loss of Echs1 protein expression was validated via Western blot analysis. Each dot represents an independent well. **(B)** Mitochondria were isolated from WT and Echs1D N2As and analyzed via native gel to quantify levels of assembled OXPHOS complexes. Each dot represents an independent well. Blots were cropped for clarity and full images are provided in Supplementary Figure S5. **(C, D)** Cells were stained with MitoTracker Red **(C)** or MitoSOX **(D)** to quantify mitochondrial membrane potential and superoxide formation, respectively. Values are mean fluorescence intensity (MFI) relative to control. Data graphed are from three independent experiments; each dot represents an individual well. **(E–J)** Oxygen consumption rate was measured using an XF96 Seahorse Analyzer. Basal respiration **(E)**, ATP production **(F)**, maximum respiration **(G)**, proton leak **(H)**, spare capacity **(I)**, and extracellular acidification rate **(J)** were calculated. Data graphed are from three independent experiments; each dot represents an individual well. Student’s t-test, *p < 0.05, **p < 0.01, ***p < 0.001, ****p < 0.0001.

To determine the effects of Echs1 deficiency on mitochondrial respiratory machinery in a neuronal cell model, we measured OXPHOS complex assembly as impaired complex formation has been reported in other ECHS1D cell models and patient fibroblasts (Burgin et al., 2023). Echs1D N2A cells exhibited an overall reduction in complex assembly as compared to WT cells, with the most significant impairments in complexes II-IV (Figure 2B). As this structural impairment would be expected to impair mitochondrial function, MitoTracker and MitoSOX staining were assessed. Compared to passage-matched WT N2A, Echs1D cells had similar mitochondrial membrane potential and superoxide production, indicating preserved mitochondrial function despite OXPHOS disruptions (Figures 2C,D). Consistent with these findings, basal respiration was also preserved in Echs1D cells, as assessed via a Seahorse flux assay (Figure 2E). Unexpectedly, ATP production, maximum respiration, and spare capacity were significantly increased in Echs1D N2A compared to WT, whereas proton leak was reduced (Figures 2F–I). In parallel, glycolytic activity, as measured by extracellular acidification rate was also significantly upregulated in Echs1D cells (Figure 2J).

To determine whether the mitochondrial respiratory phenotype observed in our Echs1D N2A cell model was attributable to Echs1 loss rather than a potential off-target effect of CRISPR editing, we treated cells with either scrambled control siRNA or siRNA targeting mouse Echs1. We first confirmed that Echs1 siRNA reduced Echs1 protein levels by more than 50% in WT cells (Supplementary Figures S2A–C). We then assessed mitochondrial respiration following Echs1 knockdown in both WT and Echs1D N2A cells. In WT cells, Echs1 siRNA significantly increased basal respiration, ATP production and maximum respiration levels compared with scrambled control siRNA, recapitulating the respiratory phenotype observed in the Echs1D cell line (Supplementary Figures S2D–F). In contrast, the already elevated levels of these parameters in Echs1D cells were not further increased by Echs1 siRNA treatment. Together these findings support that the mitochondrial respiratory phenotype observed in the Echs1D N2A cells results from loss of Echs1 and is not specific to the CRISPR-generated clone.

These data indicate that despite impaired OXPHOS complex assembly, Echs1D N2A display enhanced respiratory and energetic capacity. As similar metabolic shifts accompany neuronal differentiation (Agostini et al., 2016), we next examined whether Echs1 loss promotes neuronal maturation.

### Premature differentiation in Echs1D cells

To determine whether loss of Echs1 alters neuronal morphology and differentiation, cells were stained for tubulin to assess cell size and neurite complexity (Figures 3A–C). Echs1D N2A exhibited significantly increased nuclei size and branch length compared to WT cells (Figures 3D,E), morphological features consistent with enhanced differentiation. Expression of NeuN, a marker of mature neurons (Gusel’nikova and Korzhevskiy, 2015; Lone et al., 2016), was also significantly increased in Echs1D cells (Figures 3F,G). Together, these findings indicate that loss of Echs1 promotes neuronal differentiation in N2A cells, consistent with the metabolic and mitochondrial remodeling observed during this process (Agostini et al., 2016).

**FIGURE 3:**
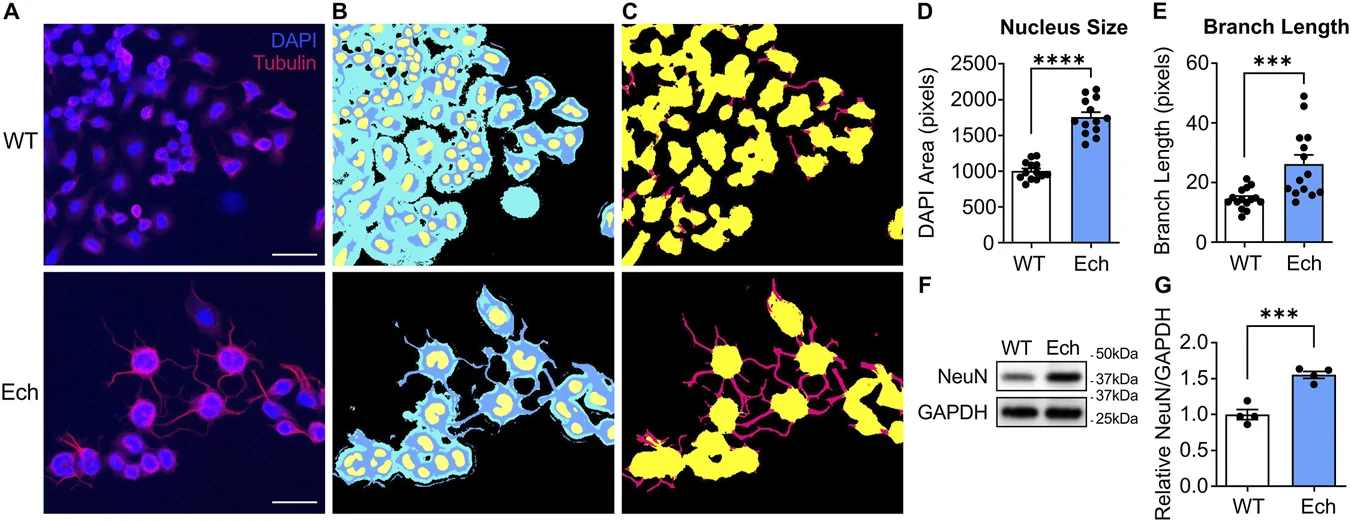
Echs1D N2As display enhanced differentiation. WT and Echs1D (Ech) N2As were stained with anti-tubulin and DAPI. **(A)** Representative images of WT (top) and Echs1D (bottom) cells. Scale bar = 50 µm. **(B)** Segmentation of nuclei performed in iLastik. Yellow mask area used as quantification of nucleus size. **(C)** Segmentation of branching performed in iLastik. Red mask area used for quantification of branch length. **(D, E)** Quantification of nuclei size **(D)** and branch length **(E)** in pixels. Data graphed are from 15 images per cell line; each dot represents the average from each image. **(F, G)** Protein was extracted from WT and Echs1D N2As and analyzed via Western blotting to measure levels of NeuN. NeuN levels were normalized to GAPDH and set relative to WT. N = 4/group. Blots were cropped for clarity and full images are provided in Supplementary Figure S6. Student’s t-test, ***p < 0.001, ****p < 0.0001.

Because neuronal differentiation is accompanied by changes in mitochondrial function, including OXPHOS complex assembly (Agostini et al., 2016), we next asked whether the assembly defects observed in Echs1D cells were a direct consequence of Echs1 loss or secondary to their enhanced differentiation state. WT and Echs1D N2A cells were treated with retinoic acid (RA) to induce differentiation, which was confirmed by increased NeuN levels in both WT and Echs1D cells (Supplementary Figure S3A). OXPHOS complex assembly was then assessed relative to DMSO-treated controls. In WT cells, RA treatment significantly increased complex I assembly, while the other OXPHOS complexes remained unchanged (Supplementary Figures S3B,C). In contrast, RA treatment of Echs1D cells further reduced OXPHOS complex assembly, exacerbating the defects already present under basal conditions. Together, these data suggest that the OXPHOS complex assembly defects observed in Echs1D N2A cells cannot be attributed solely to enhanced neuronal differentiation and are further exacerbated by additional differentiation.

As differentiation-associated metabolic remodeling may influence the mitochondrial phenotype observed in N2A cells, we next examined the consequences of Echs1 deficiency in an *in vivo* model of ECHS1D.

### Excess dietary valine in ECHS1D mice

A prior study from our lab characterized a compound heterozygous mouse model of ECHS1D carrying a patient-associated F33S variant and a knock-out allele. These mice exhibit epileptiform EEG activity and increased susceptibility to pentylenetetrazol (PTZ)-induced seizures compared to control animals (Eller et al., 2025). Because impaired Echs1 activity disrupts valine metabolism and leads to the accumulation of potentially toxic valine-derived intermediates, we hypothesized that excess dietary valine would worsen seizure susceptibility in ECHS1D mice. To test this, WT and ECHS1D breeding pairs were maintained on control or valine supplemented chow, and offspring remained on their respective diets throughout the study (Figure 4A). Valine supplementation was well tolerated, with no changes in survival (data not shown) or weight gain throughout the study (Figure 4B).

**FIGURE 4:**
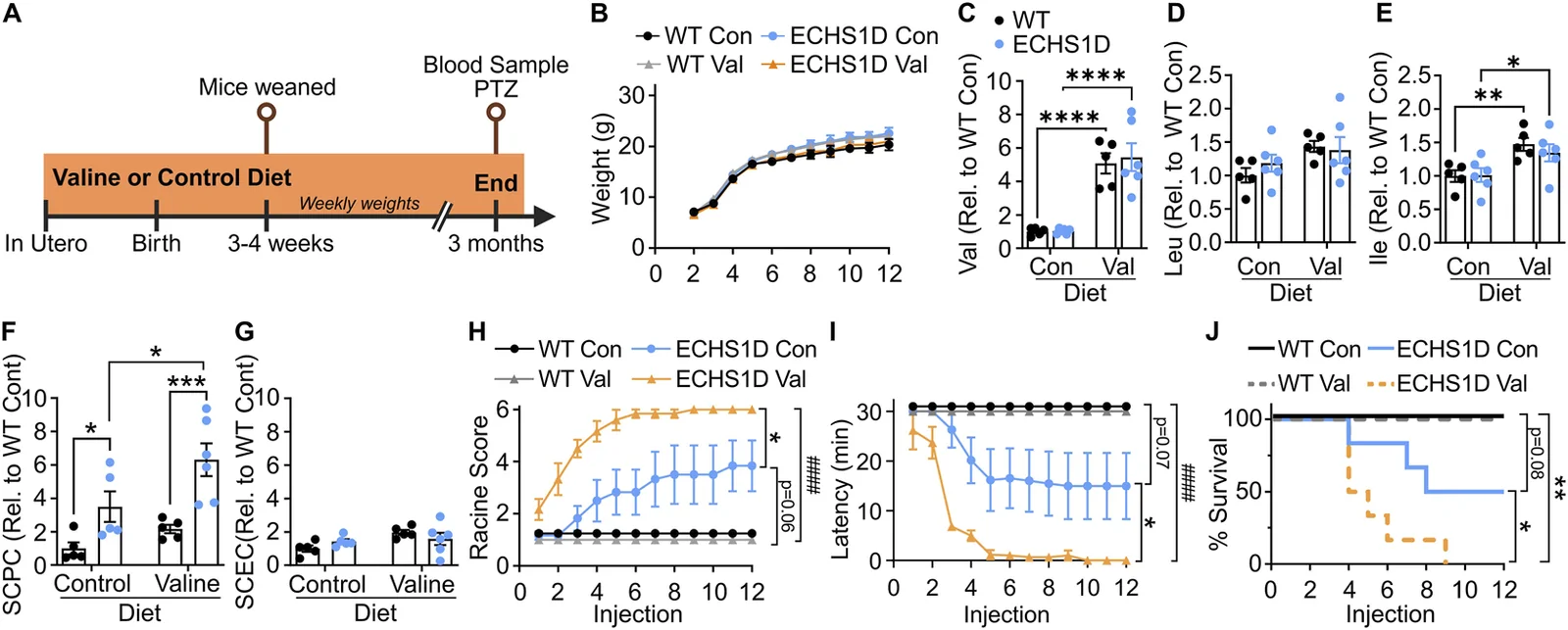
Dietary valine increases SCPC accumulation and worsens seizure susceptibility in ECHS1D mice. Breeding WT/KI and WT/KO mice were given control (Con) or valine (Val) supplemented chow, and the resulting litters were maintained on the given diet throughout the study. **(A)** Study scheme. **(B)** Weights of mice throughout study duration. **(C–G)** Concentrations of valine **(C)**, leucine **(D)**, isoleucine **(E)**, SCPC **(F)**, and SCEC **(G)** were quantified in plasma obtained at 3 months of age. Each dot represents an individual animal. **(H–J)** Mice (N = 5–6/group) were injected with PTZ (30 mg/kg) every other day for 12 days and seizure severity **(H)**, latency to seize **(I)**, and survival **(J)** was recorded following each injection. B–F: Two-way ANOVA with Uncorrected Fisher’s LSD, *p < 0.05, **p < 0.01, ***p < 0.001, ****p < 0.0001; **(G, H)** Repeat measures two-way ANOVA, Diet effect *p < 0.05, Genotype effect ^####^ p < 0.0001; **(I)** Log-rank test, *p < 0.05, **p < 0.01. Full ANOVA results are shown in Supplementary Tables S5 and S6.

At 3 months of age, circulating BCAAs and valine intermediates SCPC and SCEC were quantified in blood samples. As expected, both WT and ECHS1D mice on valine supplemented chow had significantly increased circulating valine levels (Figure 4C). Leucine levels were unaffected by genotype or diet, whereas isoleucine levels were modestly but significantly increased following valine supplementation (Figures 4D,E). Analysis of valine intermediates revealed elevated SCPC, but not SCEC, in ECHS1D mice under baseline dietary conditions (Figures 4F,G). Interestingly, valine supplementation further increased SCPC levels by approximately two-fold, whereas SCEC concentrations were unchanged despite increased valine intake. Together, these results indicate that increased dietary valine selectively promotes SCPC accumulation in the context of impaired ECHS1-dependent valine catabolism (Figures 4F,G).

Mice were then subjected to PTZ seizure induction to determine whether excess SCPC accumulation exacerbates the baseline epileptic phenotype. Consistent with our prior work (Eller et al., 2025), ECHS1D mice on control diet developed progressively severe seizures that reduced survival by 50%, while WT mice were unaffected (Figures 4H–J). Remarkably, valine supplementation significantly exacerbated seizure susceptibility in ECHS1D mice, which began seizing after the first PTZ injection and rapidly progressed in severity, culminating in 100% lethality by the ninth injection (Figures 4H–J). This effect was specific to the ECHS1D genotype, as valine supplementation did not impact seizure susceptibility in WT littermate mice, which remained seizure free throughout the protocol. Together, this data supports that increased dietary valine accelerates disease progression in ECHS1D, coinciding with enhanced SCPC accumulation and markedly worsened seizure outcomes.

### Brain metabolism following valine supplementation

Under basal conditions, ECHS1D mice exhibit largely unremarkable alterations in brain metabolism, with the exception of minor increases in alanine ratios that are suggestive of PDC dysfunction (Bedoyan et al., 2020; Eller et al., 2025; Verma et al., 2024). Given the marked exacerbation of seizure susceptibility following dietary valine supplementation, we next asked whether brain metabolism profiles are similarly altered under these conditions. At 3 months of age, a separate cohort of mice was subjected to microwave assisted fixation (Juras et al., 2023), which we found necessary to preserve *in vivo* redox state and energy charge during brain tissue collection. Brain tissues were subsequently used to quantify amino acids, energy charge, and redox status (Figure 5A). Concentrations of all amino acids are presented in Supplementary Table S2. Analysis of BCAA concentrations confirmed that dietary valine supplementation significantly increased brain valine levels in both WT and ECHS1D mice (Supplementary Figure S4A), mirroring changes observed in plasma (Figure 4C). Leucine was unaffected by diet, although ECHS1D mice on control diet had a significantly lower brain leucine concentration compared to WT controls (Supplementary Figure S4B). Similar to blood levels, brain isoleucine was mildly increased in both genotypes following valine supplementation (Supplementary Figure S4C, Figure 4E).

**FIGURE 5:**
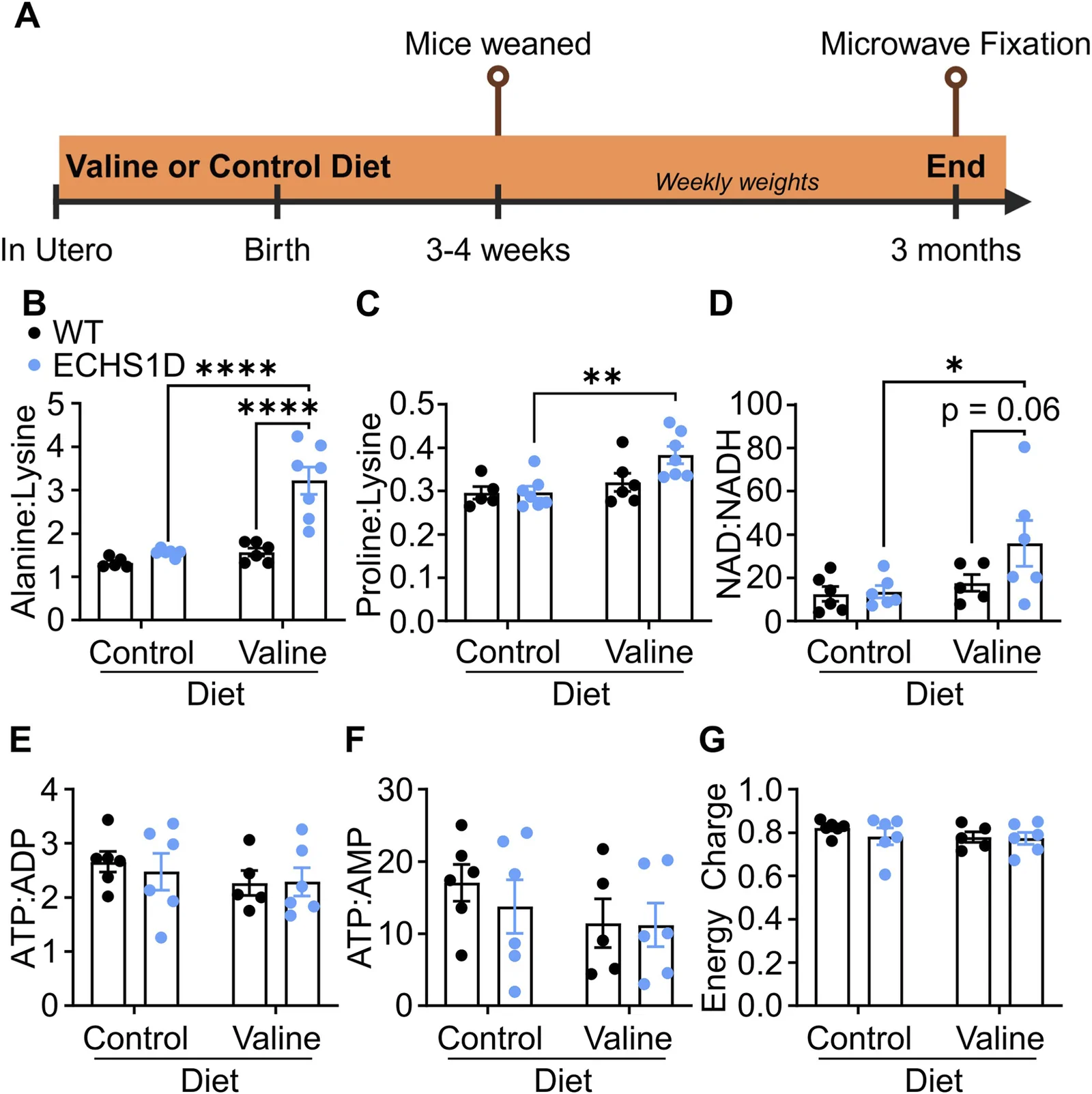
Excess dietary valine increases lysine ratios and induces oxidative imbalance without impacting energy charge. Breeding WT/KI and WT/KO mice were given control or valine supplemented chow, and the resulting litters were maintained on the given diet throughout the study. **(A)** Study scheme. **(B–F)** Ratio of alanine:lysine **(B)**, proline:lysine **(C)**, NAD:NADH **(D)**, ATP:ADP **(E)**, and ATP:AMP **(F)** was quantified in brain tissue following microwave fixation at 3 months of age. **(G)** Energy charge within the brain tissue. Each dot represents an individual animal. Two-way ANOVA with Uncorrected Fisher’s LSD, *p < 0.05, **p < 0.01, ****p < 0.0001. Full ANOVA results are shown in Supplementary Table S7.

We next analyzed the alanine and proline ratios, which are commonly used biomarkers of PDC dysfunction (Bedoyan et al., 2020; Verma et al., 2024). Relative to ECHS1D mice on control diet or WT mice on either diet, valine-supplemented ECHS1D mice showed a 2–3 fold increase in the alanine:lysine ratio, and a small but significant increase in the ratio of proline:lysine (Figures 5B,C). These findings are consistent with excess accumulation of valine-derived intermediates contributing to secondary PDC dysfunction. Notably, these ratios were not increased in ECHS1D mice maintained on a control diet despite elevated SCPC levels, suggesting a threshold of metabolite accumulation may be required to measurably impair PDC activity.

Oxidative stress as a result of decreased NADH production is common following PDC disruptions (Glushakova et al., 2011; Jelinek and Moxley, 2021). In support of this, ECHS1D mice on valine supplement had an increased NAD:NADH ratio, demonstrating an oxidative imbalance (Figure 5D). Despite this PDC impairment and subsequent NADH depletion, unexpectedly, cellular high energy nucleotides (i.e., ATP, ADP, and AMP) were maintained, suggesting energy charge deficits do not contribute to the observed phenotypes (Figures 5E–G).

### Valine restriction in ECHS1D mice

Given the role of impaired valine catabolism in ECHS1D pathogenesis, therapeutic interventions have investigated dietary valine reduction as a strategy to slow disease progression (Abdenur et al., 2020; Kuwajima et al., 2021; Pata et al., 2022; Sato-Shirai et al., 2021; Shayota et al., 2019). To model this approach, WT and ECHS1D mice were placed on an amino acid defined control diet (AA) or valine restricted diet (VR) upon weaning (Figure 6A). This age was selected to recapitulate adolescent intervention. A 67% reduction was used to mimic the ∼70% reduction that has been sufficient to improve symptoms in patients (Abdenur et al., 2020). This level of reduction has also been shown to be well-tolerated in WT mice, demonstrating feasibility (Yu et al., 2021). In agreement with this, WT and ECHS1D survival was unaffected by valine restriction (data not shown), although typical weight gain was significantly reduced in both genotypes (Figure 6B).

**FIGURE 6:**
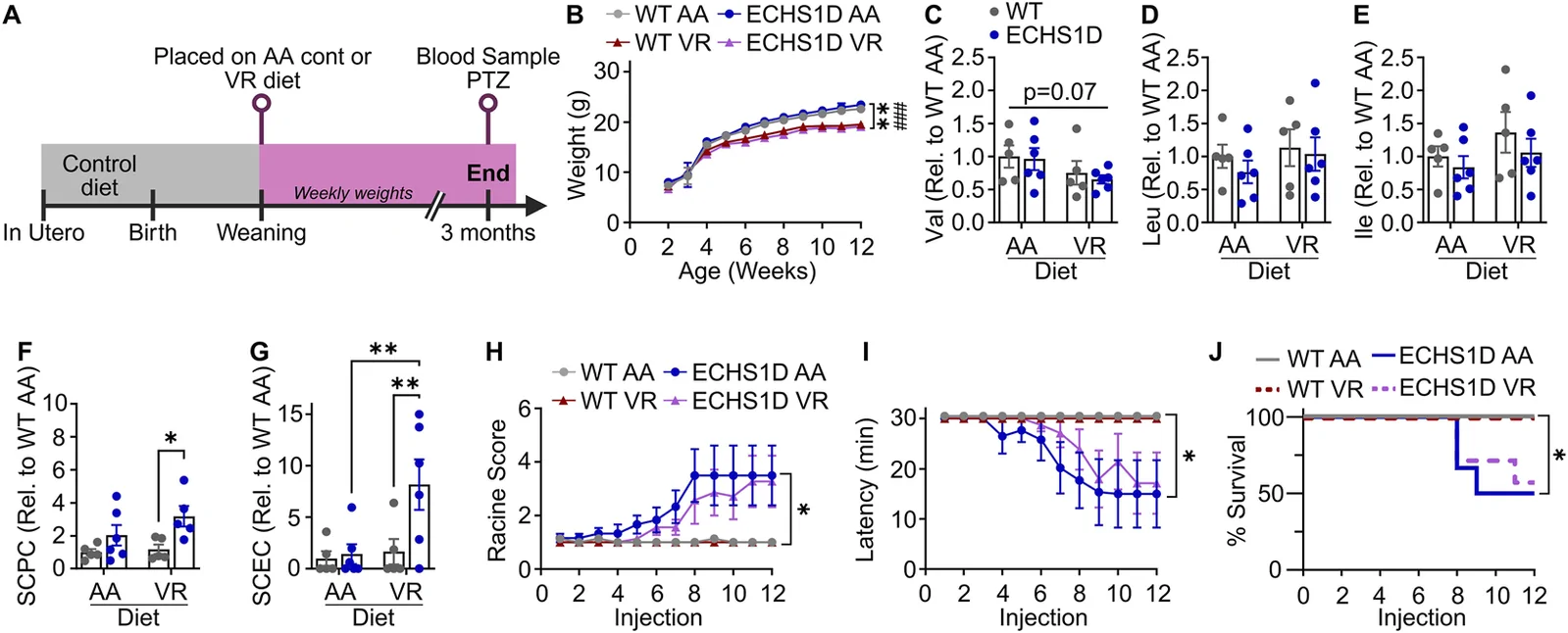
Dietary valine restriction induces SCEC accumulation and does not improve seizure susceptibility in ECHS1D mice. Breeding WT/KI and WT/KO mice were given control chow, and the resulting litters were weaned on a separate amino acid control (AA) or a valine restricted (VR) diet. **(A)** Study scheme. **(B)** Weights of mice throughout study duration. N = 8–12 mice/group. **(C–E)** Concentration of valine **(C)**, leucine **(D)**, isoleucine **(E)**, SCPC **(F)**, and SCEC **(G)** were quantified in plasma obtained at 3 months of age. Each dot represents an individual animal. **(H, I)** Mice (N = 5–6/group) received 12 injections of PTZ (30 mg/kg) every other day and seizure severity **(H)**, latency to seize **(I)**, and survival **(J)** was recorded following each injection. **(B)** Repeat measures two-way ANOVA, Diet effect ****p < 0.0001; **(C–G)** Two-way ANOVA with Uncorrected Fisher’s LSD, *p < 0.05, **p < 0.01; **(H, I)** Repeat measures two-way ANOVA, Genotype effect *p < 0.05; **(J)** Log-rank test, *p < 0.05. Full ANOVA results are shown in Supplementary Tables S9 and S10.

As in the valine supplementation study (Figure 4), blood samples were collected at 3 months of age to quantify circulating BCAAs and valine-derived intermediates. At the level of valine reduction used, circulating valine showed a downward trend in both WT and ECHS1D mice, although this decrease did not reach statistical significance (Figure 6C). Valine restriction did not significantly alter circulating leucine or isoleucine levels in either genotype (Figures 6D,E). Consistent with prior findings, ECHS1D mice maintained on the control diet showed elevated SCPC compared with WT, and this accumulation was not attenuated by valine restriction (Figure 6F). Unexpectedly, SCEC, which was not increased in the previous experimental groups (Figure 4F), was significantly elevated in ECHS1D mice subjected to valine restriction (Figure 6G). These findings suggest that valine restriction may alter additional metabolic pathways leading to aberrant SCEC production.

These mice were then subjected to PTZ-induced seizure testing to determine whether the reduction in circulating valine was sufficient to reduce seizure susceptibility, or if the increased SCEC might contribute to seizure severity in a manner similar to SCPC accumulation. All WT mice remained seizure free following PTZ challenge (Figures 6H,I). In contrast, ECHS1D mice on either control or valine restricted diet developed severe seizures, resulting in 50% mortality (Figures 6H–J). Together, these findings indicate that valine restriction alone at the level tested does not ameliorate seizure susceptibility in ECHS1D mice and may be associated with adverse outcomes in regard to increased SCEC levels.

### Brain metabolism following valine restriction

The atypical accumulation of SCEC in ECHS1D mice following valine restriction suggests that other metabolic pathways may be perturbed under these conditions. To further define brain metabolic profiles, an independent cohort of mice was subjected to microwave assisted fixation (Juras et al., 2023) for quantification of amino acids, energy charge, and redox status in brain tissue (Figure 7A). Concentrations of all measured amino acids are presented in Supplementary Table S3. Under baseline dietary conditions, the alanine:lysine ratio was not significantly increased in ECHS1D mice and was not altered by valine restriction (Figure 7B). Within the valine restricted group, ECHS1D mice had a modest but significantly significant increase in the alanine:lysine ratio relative to WT controls; however, the biological significance of this small change remains unclear (Figure 7B). Similarly, the proline:lysine ratio was modestly reduced in WT mice following valine restriction, whereas this ratio was unaffected by diet within ECHS1D mice and did not differ between genotypes under either dietary condition (Figure 7C).

**FIGURE 7:**
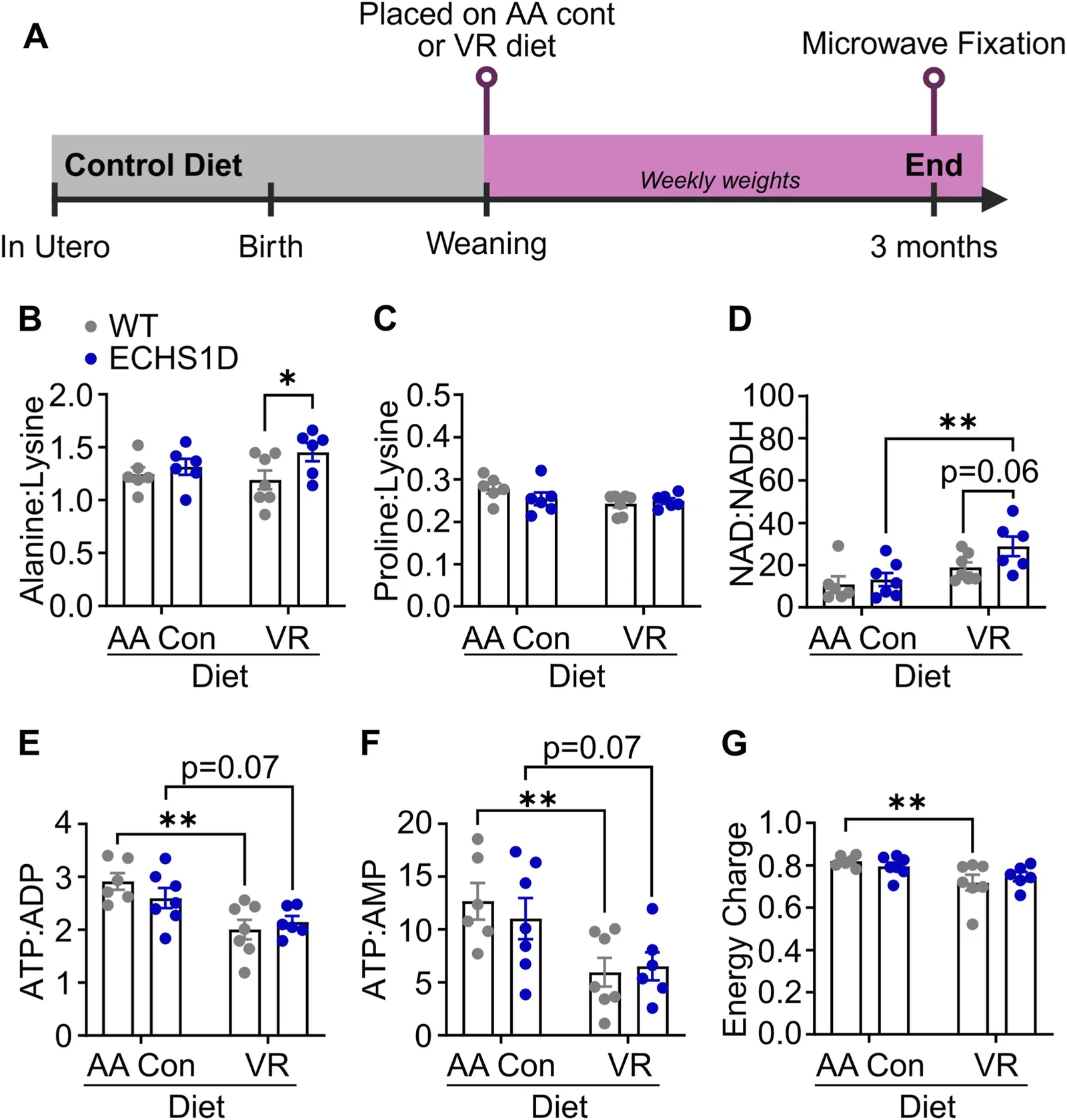
Dietary valine restriction disrupts energy charge in both WT and ECHS1D mice. At weaning, ECHS1D and WT littermates were placed on amino acid control (AA) or a valine restricted (VR) chow. **(A)** Study scheme. **(B–F)** Ratio of alanine:lysine **(B)**, proline:lysine **(C)**, NAD:NADH **(D)**, ATP:ADP **(E)**, and ATP:AMP **(F)** was quantified in brain tissue following microwave fixation at 3 months of age. **(G)** Energy charge within the brain tissue. Each dot represents an individual animal. Two-way ANOVA with Uncorrected Fisher’s LSD, *p < 0.05, **p < 0.01. Full ANOVA results are shown in Supplementary Table S11.

Consistent with previous results, the NAD:NADH ratio did not differ between WT and ECHS1D mice maintained on the control diet (Figure 7D). Interestingly, valine restriction significantly increased the NAD:NADH ratio in both genotypes, an effect similar to that observed with valine supplementation (Figure 5D). However, in contrast to valine supplementation, ATP:ADP and ATP:AMP ratios were significantly decreased in both WT and ECHS1D mice subjected to valine restriction, indicating reduced cellular energy charge independent of genotype (Figures 7E,F). However, when the total energy charge was calculated (Atkinson, 1968), only valine restricted WT mice had a statistically significant reduction (p = 0.009) while the reduction in ECHS1D mice did not reach statistical significance (p = 0.181; Figure 7G). These results indicate that a 67% reduction of dietary valine alters brain redox and energy metabolism, even in the absence of overt disease, and may introduce metabolic stress that is independent of ECHS1 genotypes.

## Discussion

In ECHS1D, impaired valine catabolism is proposed to drive disease through the accumulation of toxic, reactive intermediates capable of inhibiting the PDC and other mitochondrial enzymes, although direct experimental evidence has been limited. In the present study, we demonstrate that intracellular accumulation of SCPC is sufficient to induce cellular toxicity associated with impaired mitochondrial respiration. Moreover, we show that excess dietary valine increases SCPC accumulation in an ECHS1D mouse model, accompanied by worsened seizure susceptibility and PDC dysfunction. In contrast, 67% valine restriction starting in adolescence was insufficient to reduce SCPC accumulation and instead led to an unexpected increase in SCEC in ECHS1D mice, highlighting the complexity of valine metabolism in this preclinical model and the potential for unanticipated metabolic effects of dietary manipulation.

Consistent with the complexity of valine-derived metabolic perturbations reported in patients, we examined the combined and individual effects of ECHS1D-associated intermediates on cellular viability and mitochondrial function. Treatment of WT N2A with a pooled mixture of SCPC, SCEC and DHMB at 5 mM each significantly reduced cell viability. Among the individual intermediates, SCPC exhibited the greatest toxicity, although concentrations >15 mM were required to significantly reduce cell survival, while SCEC and DHMB alone did not impact viability at the concentrations tested. Notably, at sub-toxic doses, each metabolite exerted distinct effects on mitochondrial parameters, suggesting that ECHS1D-associated intermediates may affect mitochondrial function through multiple mechanisms. Specifically, both SCPC and DHMB increased mitochondrial membrane potential, while SCEC increased superoxide levels. Given that increased membrane potential can promote superoxide production (Korshunov et al., 1997), simultaneous exposure to multiple accumulating intermediates could potentially compound mitochondrial dysfunction. Future studies examining mitochondrial respiration following combined intermediate exposure will be important for defining these effects.

An important limitation of these experiments is that the 10–25 mM concentrations used for individual metabolite treatments exceed the concentrations reported in the urine and serum of patients with ECHS1D. In addition, the LC-MS protocol used to detect valine-derived intermediates in our cell and mouse studies enabled relative comparisons between experimental conditions but did not allow for absolute quantification. Consequently, we cannot determine how the levels of these intermediates in our ECHS1D cell and mouse models compare with those observed in patients. Reported urinary concentrations range from 5.16 to 13.96 μmol/mmol for SCPC and 1.36–3.94 μmol/mmol for SCEC, while serum concentrations range from 46.4 to 168.5 nmol/L for SCPC and 20.8–27.4 nmol/L for SCEC (Kuwajima et al., 2021). Moreover, whether circulating or urinary concentrations accurately reflect metabolite exposure within affected tissues, particularly the CNS, remains unclear. Future studies incorporating absolute quantification of valine-derived intermediates in ECHS1D cell and mouse models, together with prolonged or combined exposures at physiologically relevant concentrations, will be important for establishing their relationship to patient metabolite levels and determining how chronic metabolite accumulation contributes to mitochondrial dysfunction in ECHS1D.

Interestingly, our studies in Echs1D N2A did not recapitulate the impaired mitochondrial respiration reported in Echs1-KO osteosarcoma cells or patient fibroblasts (Burgin et al., 2023; Burgin et al., 2022). Despite significant reductions in OXPHOS complex assembly, Echs1D N2A maintained basal respiration and exhibited increased ATP production, maximal respiration, and spare respiratory capacity, together with increased glycolytic activity. siRNA-mediated knockdown of ECHS1 recapitulated the increased mitochondrial fitness seen in the ECHS1D cell line, supporting an association between Echs1 loss and these metabolic changes rather than a clone-specific effect. Furthermore, induction of neuronal differentiation with retinoic acid exacerbated rather than reproduced the OXPHOS assembly defects in Echs1D cells, indicating that impaired complex assembly cannot be attributed solely to their enhanced differentiation state.

The altered metabolic phenotype of Echs1D N2A cells was accompanied by increased neuronal differentiation, including greater neurite complexity and NeuN expression. Neuronal maturation requires substantial metabolic and mitochondrial remodeling, including increased mitophagy and altered glucose metabolism (Agostini et al., 2016; Liu et al., 2024). Such remodeling may therefore contribute to the preserved or enhanced respiratory capacity observed in Echs1D N2A cells despite impaired OXPHOS complex assembly. Together with the contrasting respiratory deficits reported in non-neuronal ECHS1-deficient models, these findings suggest that the cellular response to ECHS1 deficiency may be highly dependent on cell-type and differentiation state. This distinction may be particularly important for understanding ECHS1D, in which neurological manifestations predominate despite the broad metabolic roles of ECHS1.

Although the mechanisms linking ECHS1 loss to altered neuronal differentiation remain to be defined, transcriptional and epigenetic remodeling may contribute to this effect. Reduction of Echs1 has been shown to enhance histone lysine crotonylation (Kcr) in mouse cardiomyocytes (Tang et al., 2021), a chromatin modification that suppresses proliferation and promotes differentiation in neural stem progenitor cells (NSPCs) (Dai et al., 2021). Notably, microcephaly has been reported in a subset of individuals with ECHS1D (Ganetzky et al., 1993), a phenotype that may reflect impaired NSPC proliferation, a mechanism increasingly recognized across neurodevelopmental disorders (Shigenaka et al., 2025). Thus, premature or dysregulated neuronal differentiation represents a potential contributor to ECHS1D pathogenesis that warrants further investigation. Future studies examining epigenetic regulation, NSPC proliferation and differentiation, and neuronal maturation in ECHS1D models will be needed to determine whether these processes contribute to the neurological phenotype of the disease (Ganetzky et al., 1993; Eller et al., 2025).

The increased SCPC levels observed in ECHS1D mice under conditions of both typical and excess dietary valine intake are consistent with preferential accumulation of methacrylyl-CoA resulting from the loss of Echs1 function. In contrast, increased SCEC levels are consistent with accumulation of acryloyl-CoA, which can arise from propionyl-CoA generated through valine catabolism or through the catabolism of odd-chain fatty acids, methionine, isoleucine, and threonine (Figure 1A) (Peters et al., 2014). Because SCEC levels remained unchanged under typical and excess valine intake, despite impaired valine degradation upstream of propionyl-CoA, these alternative metabolic pathways may contribute more substantially to acryloyl-CoA production in ECHS1D. Unexpectedly, however, the valine restriction paradigm we tested significantly increased SCEC levels in ECHS1D mice. Because multiple amino acid and fatty acid catabolic pathways can contribute to propionyl-CoA production (Wongkittichote et al., 2017), this finding may suggest that reduced valine availability may drive compensatory utilization of alternative substrates (e.g., isoleucine, methionine, odd-chain fatty acids, or threonine) to sustain propionyl-CoA flux. In the context of ECHS1D, increased flux through these pathways could promote acryloyl-CoA accumulation and subsequent SCEC formation, consistent with the increase observed following valine restriction. We were unable to reliably quantify DHMB in mouse blood, limiting assessment of flux through this arm of valine metabolism *in vivo*. Collectively, these findings indicate that excess dietary valine preferentially increases the metabolic burden associated with the methacrylyl-CoA pathway in ECHS1D, whereas valine restriction may produce an unexpected increase in SCEC that may reflect altered flux through alternative propionyl-CoA-producing pathways.

Excess dietary valine in ECHS1D mice exacerbated the accumulation of SCPC and was associated with worsened seizure susceptibility and reduced PDC activity. These findings are consistent with clinical observations linking higher levels of valine-derived metabolites with more severe disease outcomes in individuals with ECHS1D (Liu et al., 2025). However, additional studies are needed to establish a direct causal relationship between specific metabolite accumulation and disease phenotypes, as increased dietary valine could perturb multiple metabolic pathways beyond toxic intermediate production. Whether SCEC or SCPC accumulation contributes to other disease-relevant phenotypes such as EEG abnormalities or inflammation sensitivity (Eller et al., 2025) also remains to be determined.

In contrast, valine restriction initiated during adolescence failed to reduce SCPC levels and did not offer protection against seizure induction in ECHS1D mice. Of note, despite increased SCEC levels, valine restriction did not worsen seizure severity in ECHS1D mice, suggesting that SCEC may not drive disease progression as directly as SCPC. Although PDC activity was unaffected, as indicated by unchanged alanine and proline ratios, valine restriction did induce oxidative imbalance and energy depletion in both WT and ECHS1D mice; these findings suggest other energy production pathways that do not involve PDC may be impaired. Furthermore, both WT and ECHS1D mice subjected to valine restriction had reduced body weight gain, consistent with prior reports in mice maintained on BCAA-restricted diets (Richardson et al., 2021; Yu et al., 2021). Whether impaired weight gain, particularly during development, could have detrimental consequences for individuals with ECHS1D remains an important unanswered question. While PTZ-induced seizure susceptibility is a surrogate measure of the epileptic phenotype in Echs1D mice (Eller et al., 2025), it does not fully recapitulate the full neurological phenotype. Future studies incorporating EEG, behavioral testing, and broader neurological phenotype will be important to more comprehensively define disease progression and therapeutic response.

Importantly, these results demonstrate that reduced valine intake has global metabolic consequences that are independent of disease context. As such, interpretation of phenotypic rescue in ECHS1D mice remains difficult without additional paradigms that sufficiently mitigate the widespread metabolism shifts. In this study, dietary intervention was performed at a single time point in adolescence. Additional studies are needed to determine if earlier treatment may offer more favorable outcomes. Further, only a single level of valine restriction was evaluated; therefore, future work should employ a titration-based approach to dietary modification, potentially including adjustment of additional amino acid components, to identify regimens that mitigate metabolite accumulation without impairing growth or inducing secondary metabolic stress.

In conclusion, our findings support a model in which accumulation of ECHS1-specific valine intermediates, particularly SCPC, is associated with mitochondrial dysfunction and correlates with disease severity in ECHS1D. Our results highlight the complexity of valine metabolic pathways and the potential for unintended consequences with dietary intervention alone. Together, these results confirm the role of impaired valine metabolism as a key contributor to disease progression and underscore the need for therapeutic strategies that directly restore metabolic function rather than relying on substrate restriction alone.

## Data Availability

The raw data supporting the conclusions of this article will be made available by the authors, without undue reservation.
