# Supplementary material for: Disrupted valine metabolism and SCPC-mediated neurological toxicity in ECHS1 deficiency
Source: Front Mol Biosci. 2026 Oct 13;13:1861051. doi: 10.3389/fmolb.2026.1861051 (PMC13710032; doi:10.3389/fmolb.2026.1861051)
Supplement: Supplementary file 1 [file DataSheet1.docx]

**Supplemental Data**

**Supplemental Figure 1: Lipofectamine does not affect cell viability but alters final SCPC levels.** WT N2A cells were treated with 25 mM of SCPC alone or with lipofectamine for 48 hours. (**A**) Percent viability was quantified using untreated N2As as control. (**B**) Relative SCPC levels were determined via LC-MS analysis. Values are relative to untreated control. Each dot represents an individual well. One-way ANOVA with Sidak’s multiple comparisons, ****p<0.0001.

**Supplemental Figure 2: ECHS1 knockdown in WT N2A cells recapitulates the mitochondrial respiratory phenotype of Echs1D N2A cells.** (**A-C**) WT N2A were treated with 40nM of control scrambled siRNA (scr) or siRNA targeting mouse *Echs1* (siRNA). Knockdown of Echs1 was confirmed via RT-qPCR for mRNA (A) or via western blot for protein (B-C). (**D-F**) WT and Echs1D N2A were treated with 40nM of scr or siRNA for 48 hours. Oxygen consumption rate was measured using an XF96 Seahorse Analyzer. Basal respiration (**C**), ATP production (**D**), and maximum respiration (**E**) were calculated. Each dot represents an individual well. (A) Student’s unpaired t-test, p****<0.0001, (D-F) Two-way ANOVA with Tukey’s multiple comparisons, *p<0.05, **p<0.01, ***p<0.001,****p<0.0001.

**Supplemental Figure 3: Retinoic acid treatment in WT N2As does not reduce OXPHOS complex assembly.** WT and Echs1D N2As were treated with DMSO or retinoic acid (RA; 20mM, dissolved in DMSO) for 48 hours. (**A**) Whole cell lysates were analyzed via western blot to evaluate the level of NeuN expression. (**B-C**) Mitochondria were isolated and analyzed via native gel to quantify levels of assembled OXPHOS complexes. Each dot represents an independent well. Two-way ANOVA with Tukey’s multiple comparisons, *p<0.05, **p<0.01, ****p<0.0001.

**Supplemental Figure 4: Brain BCAA concentrations.** Mice on control or valine supplemented diet were subjected to microwave-assisted fixation at 3 months of age and brains were collected for quantification of BCAA concentrations. (**A-C**) Concentration of valine (A), leucine (B), and isoleucine (C) in WT and ECHS1D brains. Each dot represents an individual animal. Two-way ANOVA with Uncorrected Fisher's LSD, *p<0.05, **p<0.01. Full ANOVA results are shown in Supplemental Table 8.

**Supplemental Figure 5: Uncropped blots used in Figure 2.** (**A-B**) Uncropped blots used for quantification of Echs1 (A) with GAPDH (B) used as a housekeeping protein. (**C-D**) Uncropped blots used for quantification of OXPHOS assembly. (D) Molecular weight ladder on gel that was imaged via SimplyBlue stain prior to transfer to the membrane shown in (C). Bottom blots show outline of region used for main figure. n.s. = non-specific

**Supplemental Figure 6: Uncropped blots used in Figure 3.** (**A-B**) Uncropped blots used for quantification of NeuN (A) with GAPDH (B) used as a housekeeping protein. Bottom blots show outline of region used for main figure. n.s. = non-specific

| **Diet** | **Control** | **Val Supplement** | **AA Con** | **Val Restricted** |
| --- | --- | --- | --- | --- |
| Teklad Diet Name  Teklad Diet Number  Color | 16% Protein  2916  None | 3.8% Valine  TD.220023  None | 21% Protein  TD.140711  Red | Val 2/3 Adj  TD.210282  Green |
| **Amino Acids** | **g/kg** | **g/kg** | **g/kg** | **g/kg** |
| Aspartic Acid  Glutamic Acid  Alanine  Glycine  Threonine  Proline  Serine  Leucine  Isoleucine  Valine  Phenylalanine  Tyrosine  Methionine  Cystine  Lysine  Histidine  Arginine  Tryptophan | 10.0  33.3  9.0  7.0  6.0  15.0  8.0  19.0  7.0  8.0  9.0  5.0  3.0  3.0  8.0  4.0  8.0  2.0 | 9.7  32.0  8.7  6.8  5.2  14.5  7.8  17.0  6.2  38.0  8.3  4.5  4.0  2.5  6.7  3.5  7.6  1.6 | 20.58  28.97  9.38  2.96  9.7  7.41  7.41  25.4  7.8  8.4  6.6  6.9  6.7  7.2  20.38  4.6  6.3  3.4 | 21.3844  29.859  9.9183  3.4135  9.7  8.1054  8.0448  25.4  7.8  2.74  6.6  6.9  6.7  7.2  20.38  4.6  6.3  3.4 |
| **%by weight** | **%by weight** | **%by weight** | **%by weight** | **%by weight** |
| Protein  Carbohydrates  Fat | 16.4  48.5  3.7 | 18.8  51.2  3.4 | 21.5  58.0  8.1 | 21.5  58.1  8.1 |

**Supplemental Table 1: Content of diets used in mouse studies.**  TD.220023 was generated using 2916 as a base, and TD.210282 was generated using TD.140711 as a base.

| **Amino Acid** | **WT**  **Con**  (Mean  ±SEM) | **ECH**  **Con**  (Mean  ±SEM) | **WT Val**  (Mean  ±SEM) | **ECH Val**  (Mean  ±SEM) | **WT**  **Con v. ECH**  **Con**  (p-value) | **WT Con v. WT Val**  (p-value) | **ECH**  **Con v. ECH Val**  (p-value) | **WT Val v. ECH Val**  (p-value) |
| --- | --- | --- | --- | --- | --- | --- | --- | --- |
| **Ala** | 302.9  (±4.4) | 329.4  (±14.7) | 295.1  (±26.5) | 541.2  (±52.2) | 0.61 | 0.87 | **0.0002** | **<0.0001** |
| **Arg** | 118.9  (±7.6) | 99.8  (±10.9) | 116.0  (±11.7) | 105.1  (±6.7) | 0.19 | 0.84 | 0.68 | 0.43 |
| **Asn** | 87.0  (±4.3) | 74.3  (±6.9) | 86.8  (±7.2) | 87.2  (±6.2) | 0.19 | 0.98 | 0.15 | 0.96 |
| **Asp** | 2455.5  (±131.9) | 2261.9  (±189.6) | 2443.4  (±167.5) | 2177.3  (±83.4) | 0.39 | 0.96 | 0.68 | 0.22 |
| **Cit** | 11.0  (±0.8) | 8.0  (±0.8) | 10.5  (±2.1) | 10.3  (±0.9) | 0.12 | 0.79 | 0.18 | 0.92 |
| **Gln** | 5411.9  (±186.8) | 4623.1  (±465.6) | 4768.2  (±371.0) | 5436.4  (±301.5) | 0.15 | 0.27 | 0.11 | 0.22 |
| **Glu** | 6016.2  (±189.8) | 5136.9  (±493.7) | 5392.9  (±378.0) | 5789.1  (±301.1) | 0.13 | 0.29 | 0.21 | 0.46 |
| **Gly** | 1146.8  (±83.6) | 1199.0  (±133.3) | 1191.4  (±108.3) | 1193.1  (±71.3) | 0.74 | 0.79 | 0.97 | 0.99 |
| **His** | 93.0  (±2.2) | 73.4  (±7.5) | 72.7  (±7.7) | 79.8  (±4.6) | **0.04** | **0.04** | 0.45 | 0.43 |
| **Ile** | 14.1  (±1.0) | 11.7  (±1.4) | 17.4  (±1.9) | 16.1  (±1.3) | 0.30 | 0.15 | **0.04** | 0.54 |
| **Leu** | 42.9  (±1.5) | 32.2  (±3.1) | 41.1  (±4.0) | 39.6  (±3.7) | **0.04** | 0.72 | 0.11 | 0.74 |
| **Lys** | 230.2  (±11.6) | 176.5  (±18.9) | 190.5  (±18.3) | 171.1  (±10.6) | **0.03** | 0.11 | 0.80 | 0.39 |
| **Met** | 68.2  (±4.0) | 52.7  (±3.8) | 40.6  (±5.1) | 39.4  (±2.6) | **0.02** | **0.0001** | **0.02** | 0.83 |
| **Orn** | 10.5  (±0.6) | 7.8  (±0.9) | 9.4  (±1.0) | 10.1  (±0.3) | **0.02** | 0.33 | **0.04** | 0.52 |
| **Phe** | 49.5  (±3.1) | 42.9  (±4.5) | 48.5  (±8.9) | 46.9  (±3.1) | 0.42 | 0.91 | 0.59 | 0.83 |
| **Pro** | 67.6  (±2.2) | 57.0  (±1.4) | 60.5  (±5.6) | 69.1  (±1.4) | **0.04** | 0.15 | **0.01** | 0.07 |
| **Ser** | 730.5  (±42.2) | 606.6  (±72.1) | 603.1  (±52.1) | 856.2  (±69.7) | 0.2 | 0.2 | **0.008** | **0.01** |
| **Thr** | 304.7  (±13.8) | 264.3  (±26.1) | 211.9  (±21.7) | 251.0  (±17.2) | 0.21 | **0.009** | 0.64 | 0.20 |
| **Tyr** | 64.3  (±4.4) | 52.3  (±7.2) | 60.1  (±14.9) | 54.1  (±4.9) | 0.36 | 0.75 | 0.88 | 0.63 |
| **Val** | 54.1  (±2.5) | 42.9  (±6.0) | 160.8  (±31.2) | 110.1  (±11.3) | 0.65 | **0.0004** | **0.006** | **0.04** |

**Supplemental Table 2: Average brain amino acid concentrations and p-values following valine supplementation.** Mice maintained on control or valine-supplemented diets underwent microwave-assisted fixation at 3 months of age and brain amino acids were quantified via LC-MS/MS. Values represent mean amino acid concentrations in nmol/g of tissue ± SEM. Statistical comparisons were performed using two-ANOVA with uncorrected Fisher’s LSD; significant values (p < 0.05) are shown in bold. N = 5-7 mice per group.

| **Amino Acid** | **WT Con** (Mean  ±SEM) | **ECH Con** (Mean  ±SEM) | **WT VR**  (Mean  ±SEM) | **ECH VR**  (Mean  ±SEM) | **WT**  **Con v. ECH**  **Con**  (p-value) | **WT**  **Con v.WT VR**  (p-value) | **ECH**  **Con v. ECH VR**  (p-value) | **WT Val v. ECH VR**  (p-value) |
| --- | --- | --- | --- | --- | --- | --- | --- | --- |
| **Ala** | 307.4  (±32.5) | 317.5  (±33.3) | 244.2  (±14.6) | 344.9  (±32.4) | 0.81 | 0.12 | 0.51 | **0.02** |
| **Arg** | 78.4  (±2.8) | 74.0  (±5.4) | 69.7  (±3.5) | 72.3  (±6.6) | 0.53 | 0.20 | 0.80 | 0.70 |
| **Asn** | 102.1  (±15.0) | 90.7  (±6.3) | 77.8  (±3.1) | 90.7  (±6.0) | 0.36 | **0.05** | 0.99 | 0.28 |
| **Asp** | 3407.3  (±148.5) | 3221.0  (±202.2) | 2845.5  (±147.3) | 3341.2  (±274.7) | 0.52 | **0.05** | 0.68 | 0.08 |
| **Cit** | 12.3  (±0.7) | 13.4  (±1.5) | 9.3  (±0.6) | 11.0  (±0.9) | 0.42 | **0.03** | 0.09 | 0.21 |
| **Gln** | 5282.9  (±227.8) | 5130.8  (±551.5) | 4298.8  (±191.6) | 5078.8  (±348.6) | 0.76 | **0.05** | 0.92 | 0.12 |
| **Glu** | 5730.9  (±232.5) | 5109.7  (±401.7) | 4810.7  (±151.3) | 5311.8  (±421.3) | 0.18 | **0.05** | 0.66 | 0.26 |
| **Gly** | 1246.9  (±122.6) | 1250.8  (±217.5) | 1102.3  (±102.0) | 1346.0  (±130.9) | 0.99 | 0.49 | 0.66 | 0.25 |
| **His** | 84.3  (±4.1) | 82.6  (±10.0) | 75.2  (±4.1) | 87.7  (±5.5) | 0.85 | 0.31 | 0.57 | 0.17 |
| **Ile** | 17.0  (±2.4) | 12.4  (±1.7) | 12.8  (±1.3) | 13.0  (±1.4) | 0.08 | 0.09 | 0.82 | 0.94 |
| **Leu** | 46.8  (±8.6) | 37.4  (±5.1) | 35.2  (±1.9) | 35.0  (±3.9) | 0.23 | 0.13 | 0.76 | 0.99 |
| **Lys** | 244.6  (±13.9) | 237.6  (±13.2) | 207.9  (±10.6) | 237.4  (±16.8) | 0.73 | 0.07 | 0.99 | 0.13 |
| **Met** | 56.8  (±3.8) | 57.9  (±4.6) | 45.9  (±4.4) | 43.8  (±6.1) | 0.87 | 0.12 | **0.05** | 0.75 |
| **Orn** | 5.5  (±1.0) | 8.5  (±2.4) | 14.4  (±4.7) | 8.1  (±1.8) | 0.51 | **0.05** | 0.93 | 0.15 |
| **Phe** | 51.7  (±1.8) | 48.4  (±8.1) | 47.0  (±3.6) | 53.5  (±4.9) | 0.66 | 0.51 | 0.50 | 0.37 |
| **Pro** | 68.2  (±4.8) | 60.9  (±5.6) | 50.1  (±1.6) | 59.0  (±3.8) | 0.23 | **0.004** | 0.75 | 0.13 |
| **Ser** | 630.2  (±36.7) | 615.2  (±90.2) | 575.3  (±28.6) | 751.6  (±67.1) | 0.86 | 0.51 | 0.12 | **0.04** |
| **Thr** | 451.8  (±41.6) | 462.3  (±31.8) | 414.6  (±15.9) | 500.0  (±28.6) | 0.81 | 0.38 | 0.39 | **0.05** |
| **Tyr** | 53.8  (±7.4) | 52.6  (±9.5) | 45.2  (±5.5) | 50.3  (±7.4) | 0.92 | 0.42 | 0.83 | 0.62 |
| **Val** | 61.2  (±5.5) | 42.7  (±4.0) | 35.8  (±4.9) | 42.1  (±4.8) | **0.02** | **0.001** | 0.94 | 0.36 |

**Supplemental Table 3: Average brain amino acid concentrations and p-values following valine restriction.** Mice maintained on control or valine restricted diets underwent microwave-assisted fixation at 3 months of age and brain amino acids were quantified via LC-MS/MS. Values represent mean amino acid concentrations in nmol/g of tissue ± SEM. Statistical comparisons were performed using two-ANOVA with uncorrected Fisher’s LSD; significant values (p < 0.05) are shown in bold. N = 5-7 mice per group.

| Assessment | F value | P value | P value   summary | Statistically   significant   (P < 0.05)? |
| --- | --- | --- | --- | --- |
| SCPC Viability | 112.5 | <0.0001 | **** | Yes |
| SCEC Viability | 3.752 | 0.0598 | ns | No |
| DHMB Viability | 8.315 | 0.0077 | ** | Yes |
| Membrane Potential | 2.222 | 0.1046 | ns | No |
| Superoxides | 8.454 | 0.0003 | *** | Yes |
| Basal Respiration | 39.98 | <0.0001 | **** | Yes |
| ATP Production | 49.38 | <0.0001 | **** | Yes |
| Max Respiration | 14.06 | <0.0001 | **** | Yes |
| Proton Leak | 17.53 | <0.0001 | **** | Yes |
| Spare Capacity | 1.451 | 0.2356 | ns | No |

**Supplemental Table 4. One-way ANOVA statistical analysis for experiments presented in Figure 1.**

| Assessment | Source of variation | % of total variation | P value | P value   summary | Statistically   significant   (P < 0.05)? | F (DFn, DFd) |
| --- | --- | --- | --- | --- | --- | --- |
| Valine | Interaction | 0.1263 | 0.7523 | ns | No | F (1, 18) = 0.1027 |
|  | Diet | 76.38 | <0.0001 | **** | Yes | F (1, 18) = 62.15 |
|  | Genotype | 0.1627 | 0.7202 | ns | No | F (1, 18) = 0.1324 |
| Leucine | Interaction | 2.912 | 0.4193 | ns | No | F (1, 18) = 0.6832 |
|  | Diet | 20.78 | 0.0404 | * | Yes | F (1, 18) = 4.867 |
|  | Genotype | 0.8086 | 0.6683 | ns | No | F (1, 18) = 0.1897 |
| Isoleucine | Interaction | 1.276 | 0.5289 | ns | No | F (1, 18) = 0.4123 |
|  | Diet | 43.21 | 0.0015 | ** | Yes | F (1, 18) = 13.97 |
|  | Genotype | 0.8232 | 0.6122 | ns | No | F (1, 18) = 0.2661 |
| SCPC | Interaction | 2.595 | 0.2843 | ns | No | F (1, 18) = 1.222 |
|  | Diet | 15.06 | 0.0164 | * | Yes | F (1, 18) = 7.094 |
|  | Genotype | 42.18 | 0.0003 | *** | Yes | F (1, 18) = 19.87 |
| SCEC | Interaction | 10.83 | 0.1219 | ns | No | F (1, 18) = 2.651 |
|  | Diet | 20.95 | 0.0369 | * | Yes | F (1, 18) = 5.131 |
|  | Genotype | 0.02854 | 0.9344 | ns | No | F (1, 18) = 0.006989 |

**Supplemental Table 5. Two-way ANOVA model statistical analysis for experiments presented in Figure 4.**

| Assessment | Fixed effects   (type III) | P value | P value   summary | Statistically   significant   (P < 0.05)? | F (DFn, DFd) | Geisser-Greenhouse's   epsilon |
| --- | --- | --- | --- | --- | --- | --- |
| Weight | Age | <0.0001 | **** | Yes | F (1.380, 563.99) = 682.3 | 0.1380 |
|  | Group | 0.2675 | ns | No | F (3, 40) = 1.364 |  |
|  | Age x Group | 0.6725 | ns | No | F (30, 384) = 0.8662 |  |
| Racine Score – WT Con vs. ECHS1D Con. | Injection | 0.0519 | ns | No | F (1.518, 13.66) = 4.008 | 0.1380 |
|  | Genotype | 0.0618 | ns | No | F (1, 9) = 4.548 |  |
|  | Injection x Genotype | <0.0001 | **** | Yes | F (11, 99) = 4.008 |  |
| Racine Score – WT Val vs. ECHS1D Val. | Injection | <0.0001 | **** | Yes | F (2.371, 21.12) = 19.41 | 0.2155 |
|  | Genotype | <0.0001 | **** | Yes | F (1, 9) = 583.6 |  |
|  | Injection x Genotype | <0.0001 | **** | Yes | F (11, 98) = 19.41 |  |
| Racine Score – ECHS1D Con vs. ECHS1D Val. | Injection | <0.0001 | **** | Yes | F (1.829, 18.13) = 18.33 | 0.1663 |
|  | Diet | 0.0132 | * | Yes | F (1, 10) = 9.041 |  |
|  | Injection x Diet | 0.4737 | ns | No | F (11, 109) = 0.9748 |  |
| Latency - WT Con vs. ECHS1D Con. | Injection | 0.0776 | ns | No | F (1.249, 11.25) = 3.594 | 0.1136 |
|  | Genotype | 0.0752 | ns | No | F (1, 9) = 4.044 |  |
|  | Injection x Genotype | 0.0003 | *** | Yes | F (11, 99) 3.594 |  |
| Latency - WT Val vs. ECHS1D Val. | Injection | <0.0001 | **** | Yes | F (1.595, 14.21) = 34.70 | 0.1450 |
|  | Genotype | <0.0001 | **** | Yes | F (1, 9) = 796.2 |  |
|  | Injection x Genotype | <0.0001 | **** | Yes | F (11, 98) = 34.70 |  |
| Latency - ECHS1D Con vs. ECHS1D Val. | Injection | 0.0002 | *** | Yes | F (1.345, 13.33) = 21.98 | 0.1223 |
|  | Diet | 0.0193 | * | Yes | F (1, 10) = 7.759 |  |
|  | Injection x Diet | 0.0681 | ns | No | F (11, 109) = 1.769 |  |

**Supplemental Table 6. Mixed-effects model statistical analysis for experiments presented in Figure 4.**

| Assessment | Source of variation | % of total variation | P value | P value   summary | Statistically   significant   (P < 0.05)? | F (DFn, DFd) |
| --- | --- | --- | --- | --- | --- | --- |
| Alanine:Lysine | Interaction | 15.17 | 0.0018 | ** | Yes | F (1, 20) = 12.92 |
|  | Diet | 26.91 | 0.0001 | *** | Yes | F (1, 20) = 22.92 |
|  | Genotype | 27.35 | 0.0001 | *** | Yes | F (1, 20) = 23.29 |
| Proline:Lysine | Interaction | 7.593 | 0.1055 | ns | No | F (1, 21) = 2.861 |
|  | Diet | 8.355 | 0.0905 | ns | No | F (1, 21) = 3.148 |
|  | Genotype | 24.89 | 0.0059 | ** | Yes | F (1, 21) = 9.381 |
| NAD:NADH | Interaction | 6.703 | 0.1835 | ns | No | F (1, 19) = 1.906 |
|  | Diet | 16.69 | 0.0422 | * | Yes | F (1, 19) = 4.745 |
|  | Genotype | 8.281 | 0.1414 | ns | No | F (1, 19) = 2.354 |
| ATP:ADP | Interaction | 0.7588 | 0.6981 | ns | No | F (1, 19) = 0.1551 |
|  | Diet | 5.835 | 0.2884 | ns | No | F (1, 19) = 1.193 |
|  | Genotype | 0.4350 | 0.7688 | ns | No | F (1, 19) = 0.08894 |
| ATP:AMP | Interaction | 1.078 | 0.6380 | ns | No | F (1, 19) = 0.2287 |
|  | Diet | 7.707 | 0.2164 | ns | No | F (1, 19) = 1.635 |
|  | Genotype | 1.445 | 0.5863 | ns | No | F (1, 19) = 0.3064 |
| Energy charge | Interaction | 1.800 | 0.5475 | ns | No | F (1, 19) = 0.3751 |
|  | Diet | 3.743 | 0.3882 | ns | No | F (1, 19) = 0.7799 |
|  | Genotype | 2.947 | 0.4430 | ns | No | F (1, 19) = 0.6139 |

**Supplemental Table 7. Two-way ANOVA model statistical analysis for experiments presented in Figure 5.**

| Assessment | Source of variation | % of total variation | P value | P value   summary | Statistically   significant   (P < 0.05)? | F (DFn, DFd) |
| --- | --- | --- | --- | --- | --- | --- |
| Valine | Interaction | 2.624 | 0.2525 | ns | No | F (1, 21) = 1.384 |
|  | Diet | 50.98 | <0.0001 | **** | Yes | F (1, 21) = 26.90 |
|  | Genotype | 6.461 | 0.0790 | ns | No | F (1, 21) = 3.409 |
| Leucine | Interaction | 6.877 | 0.1864 | ns | No | F (1, 21) = 1.866 |
|  | Diet | 2.531 | 0.4166 | ns | No | F (1, 21) = 0.6868 |
|  | Genotype | 12.28 | 0.0822 | ns | No | F (1, 21) = 3.333 |
| Isoleucine | Interaction | 0.4094 | 0.7306 | ns | No | F (1, 21) = 0.1217 |
|  | Diet | 22.09 | 0.0181 | * | Yes | F (1, 21) = 6.570 |
|  | Genotype | 4.902 | 0.2407 | ns | No | F (1, 21) = 1.458 |

**Supplemental Table 8. Two-way ANOVA model statistical analysis for experiments presented in Supplemental Figure 3.**

| Assessment | Source of variation | % of total variation | P value | P value   summary | Statistically   significant   (P < 0.05)? | F (DFn, DFd) |
| --- | --- | --- | --- | --- | --- | --- |
| Valine | Interaction | 0.1914 | 0.8400 | ns | No | F (1, 18) = 0.04198 |
|  | Diet | 16.27 | 0.0751 | ns | No | F (1, 18) = 3.570 |
|  | Genotype | 1.035 | 0.6394 | ns | No | F (1, 18) = 0.2271 |
| Leucine | Interaction | 0.5029 | 0.7578 | ns | No | F (1, 18) = 0.09804 |
|  | Diet | 4.043 | 0.3864 | ns | No | F (1, 18) = 0.7881 |
|  | Genotype | 2.823 | 0.4677 | ns | No | F (1, 18) = 0.5504 |
| Isoleucine | Interaction | 0.5369 | 0.7406 | ns | No | F (1, 18) = 0.1130 |
|  | Diet | 8.649 | 0.1939 | ns | No | F (1, 18) = 1.821 |
|  | Genotype | 5.627 | 0.2908 | ns | No | F (1, 18) = 1.185 |
| SCPC | Interaction | 0.6696 | 0.6946 | ns | No | F (1, 18) = 0.1591 |
|  | Diet | 2.191 | 0.4798 | ns | No | F (1, 18) = 0.5208 |
|  | Genotype | 21.16 | 0.0377 | * | Yes | F (1, 18) = 5.030 |
| SCEC | Interaction | 11.24 | 0.0706 | ns | No | F (1, 18) = 3.693 |
|  | Diet | 16.62 | 0.0312 | * | Yes | F (1, 18) = 5.461 |
|  | Genotype | 14.60 | 0.0419 | * | Yes | F (1, 18) = 4.796 |

**Supplemental Table 9. Two-way ANOVA model statistical analysis for experiments presented in Figure 6.**

| Assessment | Fixed effects   (type III) | P value | P value   summary | Statistically  significant   (P < 0.05)? | F (DFn, DFd) | Geisser-Greenhouse's epsilon |
| --- | --- | --- | --- | --- | --- | --- |
| Weight – WT AA vs. WT VR | Age | <0.0001 | **** | Yes | F (2.420, 44.17) = 118.5 | 0.3025 |
|  | Diet | 0.0014 | ** | Yes | F (1, 19) = 14.04 |  |
|  | Age x Diet | 0.0030 | ** | Yes | F (8, 146) = 3.084 |  |
| Weight – ECHS1D AA vs. ECHS1D VR | Age | <0.0001 | **** | Yes | F (1.918, 31.17) = 106.0 | 0.2398 |
|  | Diet | 0.0004 | *** | Yes | F (1, 17) = 18.89 |  |
|  | Age x Diet | 0.0002 | *** | Yes | F (8, 130) = 4.174 |  |
| Racine Score – WT AA vs. ECHS1D AA. | Injection | 0.0286 | * | Yes | F (1.312, 14.43) = 5.341 | 0.1193 |
|  | Genotype | 0.0339 | * | Yes | F (1, 11) = 5.864 |  |
|  | Injection x Genotype | <0.0001 | **** | Yes | F (11, 121) = 5.481 |  |
| Racine Score – WT VR vs. ECHS1D VR. | Injection | 0.1017 | ns | No | F (1.397, 13.97) = 2.895 | 0.1270 |
|  | Genotype | 0.0917 | ns | No | F (1, 10) = 3.479 |  |
|  | Injection x Genotype | 0.0022 | ** | Yes | F (11, 110) = 2.895 |  |
| Racine Score – ECHS1D AA vs. ECHS1D VR. | Injection | 0.0063 | ** | Yes | F (1.349, 14.84) = 8.714 | 0.1226 |
|  | Diet | 0.5561 | ns | No | F (1, 11) = 0.3685 |  |
|  | Injection x Diet | 0.9985 | ns | No | F (11, 121) = 0.1761 |  |
| Latency - WT AA vs. ECHS1D AA. | Injection | 0.0327 | * | Yes | F (1.594, 17.53) = 4.533 | 0.1449 |
|  | Genotype | 0.0365 | * | Yes | F (1, 11) = 5.664 |  |
|  | Injection x Genotype | <0.0001 | **** | Yes | F (11, 121) = 4.533 |  |
| Latency - WT VR vs. ECHS1D VR. | Injection | 0.1335 | ns | No | F (1.573, 15.73) = 2.375 | 0.1430 |
|  | Genotype | 0.1264 | ns | No | F (1, 10) = 2.781 |  |
|  | Injection x Genotype | 0.0112 | * | Yes | F (11, 110) = 2.375 |  |
| Latency - ECHS1D AA vs. ECHS1D VR. | Injection | 0.0073 | ** | Yes | F (1.680, 18.48) = 7.046 | 0.1527 |
|  | Diet | 0.4896 | ns | No | F (1, 11) = 0.5110 |  |
|  | Injection x Diet | 0.9831 | ns | No | F (11, 121) = 0.3079 |  |

**Supplemental Table 10. Mixed-effects model statistical analysis for experiments presented in Figure 6.**

| Assessment | Source of variation | % of total variation | P value | P value   summary | Statistically   significant   (P < 0.05)? | F (DFn, DFd) |
| --- | --- | --- | --- | --- | --- | --- |
| Alanine:Lysine | Interaction | 5.255 | 0.2460 | ns | No | F (1, 21) = 1.425 |
|  | Diet | 0.9937 | 0.6092 | ns | No | F (1, 21) = 0.2694 |
|  | Genotype | 16.00 | 0.0497 | * | Yes | F (1, 21) = 4.337 |
| Proline:Lysine | Interaction | 7.489 | 0.1679 | ns | No | F (1, 21) = 2.040 |
|  | Diet | 12.84 | 0.0744 | ns | No | F (1, 21) = 3.526 |
|  | Genotype | 2.417 | 0.4262 | ns | No | F (1, 21) = 0.6585 |
| NAD:NADH | Interaction | 3.424 | 0.2745 | ns | No | F (1, 22) = 1.256 |
|  | Diet | 30.66 | 0.0029 | ** | Yes | F (1, 22) = 11.25 |
|  | Genotype | 8.152 | 0.0978 | ns | No | F (1, 22) = 2.990 |
| ATP:ADP | Interaction | 4.535 | 0.1932 | ns | No | F (1, 22) = 1.801 |
|  | Diet | 39.96 | 0.0006 | *** | Yes | F (1, 22) = 15.87 |
|  | Genotype | 0.6784 | 0.6089 | ns | No | F (1, 22) = 0.2694 |
| ATP:AMP | Interaction | 1.308 | 0.5097 | ns | No | F (1, 22) = 0.4492 |
|  | Diet | 34.60 | 0.0023 | ** | Yes | F (1, 22) = 11.88 |
|  | Genotype | 0.3276 | 0.7405 | ns | No | F (1, 22) = 0.1125 |
| Energy charge | Interaction | 3.432 | 0.3043 | ns | No | F (1, 22) = 1.106 |
|  | Diet | 27.95 | 0.0066 | ** | Yes | F (1, 22) = 9.008 |
|  | Genotype | 0.03733 | 0.9137 | ns | No | F (1, 22) = 0.01203 |

**Supplemental Table 11. Two-way ANOVA model statistical analysis for experiments presented in Figure 7.**
